# Apparent self-heating of individual upconverting nanoparticle thermometers

**DOI:** 10.1038/s41467-018-07361-0

**Published:** 2018-11-21

**Authors:** Andrea D. Pickel, Ayelet Teitelboim, Emory M. Chan, Nicholas J. Borys, P. James Schuck, Chris Dames

**Affiliations:** 1 Department of Mechanical Engineering, University of California, Berkeley, CA 94720 USA; 2 The Molecular Foundry, Lawrence Berkeley National Laboratory, Berkeley, CA 94720 USA; 3 Department of Mechanical Engineering, Columbia University, New York, NY 10027 USA

## Abstract

Individual luminescent nanoparticles enable thermometry with sub-diffraction limited spatial resolution, but potential self-heating effects from high single-particle excitation intensities remain largely uninvestigated because thermal models predict negligible self-heating. Here, we report that the common “ratiometric” thermometry signal of individual NaYF_4_:Yb^3+^,Er^3+^ nanoparticles unexpectedly increases with excitation intensity, implying a temperature rise over 50 K if interpreted as thermal. Luminescence lifetime thermometry, which we demonstrate for the first time using individual NaYF_4_:Yb^3+^,Er^3+^ nanoparticles, indicates a similar temperature rise. To resolve this apparent contradiction between model and experiment, we systematically vary the nanoparticle’s thermal environment: the substrate thermal conductivity, nanoparticle-substrate contact resistance, and nanoparticle size. The apparent self-heating remains unchanged, demonstrating that this effect is an artifact, not a real temperature rise. Using rate equation modeling, we show that this artifact results from increased radiative and non-radiative relaxation from higher-lying Er^3+^ energy levels. This study has important implications for single-particle thermometry.

## Abstract (web summary)

Nanoparticles are often used as nanothermometers by measuring their luminescence from upconverted energy under illumination. The authors uncover the artificial appearance of a temperature rise at high excitation intensities due to effects involving higher energy states.

## Introduction

Lanthanide-doped upconverting nanoparticles (UCNPs) are popular non-contact luminescent thermometers due to their excellent photostability^1^, good thermal sensitivity allowing for sub-1 K temperature resolution^2,3^, and biocompatibility^4,5^. UCNP thermometry has been applied in a wide variety of fields, including integrated circuit devices^6^, Brownian motion^7^, antibacterial treatment^8^, cancer therapies^9^, anti-counterfeiting^10^, and optical rotation^11^. The vast majority of UCNP-based temperature measurements have employed nanoparticle ensembles^12–14^. Consequently, the spatial resolution of these measurements is governed by the diffraction limit of the exciting laser beam. To access smaller length scales, single nanoparticle measurements^15–18^ provide spatial resolution determined by the nanoparticle size, which can be much smaller than the diffraction limit. Such single-particle measurements can thus achieve the high spatial resolution necessary to measure nanoscale hotspots present in semiconductor devices^19^, heat-assisted magnetic recording drives^20^, individual cells^21^, and nanostructures of fundamental thermal interest^22^.

A key distinction between single-particle and ensemble measurements is that detecting single particles traditionally requires excitation intensities orders of magnitude higher than those used for ensembles (10^5^–10^6^ W cm^−2^ for single particles^17,23^ compared with 1–100 W cm^−2^ for ensembles^24^). Although core–shell architectures can significantly lower the excitation intensities required for single-particle imaging^25^, reductions in absolute emission intensity at elevated temperatures^26^ often require higher excitation intensities for thermometry. Additionally, certain operating modalities, such as simultaneous optical trapping and thermometry^17,27^ require high intensities to trap individual UCNPs. Furthermore, the artifacts we measure persist at lower intensities and, though comparatively small, degrade the measurement accuracy when the magnitude of the apparent self-heating is comparable to the temperature resolution of the measurement. Thus, to elucidate the nature of these artifacts, we extend our measurements to excitation intensities of ~10^5^ W cm^−2^ where the apparent self-heating is much more pronounced. At the ensemble level, it has been shown that the luminescence intensity ratio thermometry signal is independent of excitation intensity^28^. The potential for self-heating effects at single-particle excitation intensities has received limited attention. However, recent results for individual ~1 μm particles optically trapped in solution indicate that significant (~20 K) laser-induced heating of the nanoparticle^27^ or trapping medium^29^ may occur.

In this work, we study faceted hexagonal 50 × 50 × 50 nm^3^ and cylindrical 20 × 20 × 40  nm^3^ NaYF_4_ particles doped with 20% Yb^3+^ and 2% Er^3+^, the most common UCNP composition. These nanoparticles are placed on solid substrates and primarily surrounded by air, which provides more thermal resistance than common optical trapping solvents. Nonetheless, conservative thermal estimates still predict negligible self-heating for these smaller particles compared to the ~1 μm particles in liquid: reduced absorption due to smaller particle volumes dominates the increased thermal resistance, even if sub-continuum effects that reduce thermal conductivity are considered. In sharp contrast with a conservative thermal estimate, we show that increasing the excitation intensity causes an apparent temperature rise of more than 50 K as measured by two independent thermometry methods. However, this effect is remarkably insensitive to systematic manipulation of the relevant heat dissipation pathways or the number of absorbing Yb^3+^ ions. The consistent invariance of the apparent temperature rise excludes scenarios in which large external thermal resistances limit heat dissipation from the nanoparticle. Instead, we use established rate equation models to show that a similar increase in the luminescence intensity ratio at fixed temperature is expected due to both increased multiphonon relaxation from higher-lying Er^3+^ energy levels that become more heavily populated at higher *I*_exc_ and additional green photon-emitting transitions originating from highly excited Er^3+^ states. The modeled relative increase in *r* is similar to our experimental results, confirming that such intensity-dependent photophysics, rather than thermal effects, is responsible for the apparent temperature rise.

## Results

### Experimental design

**Fig. 1:**
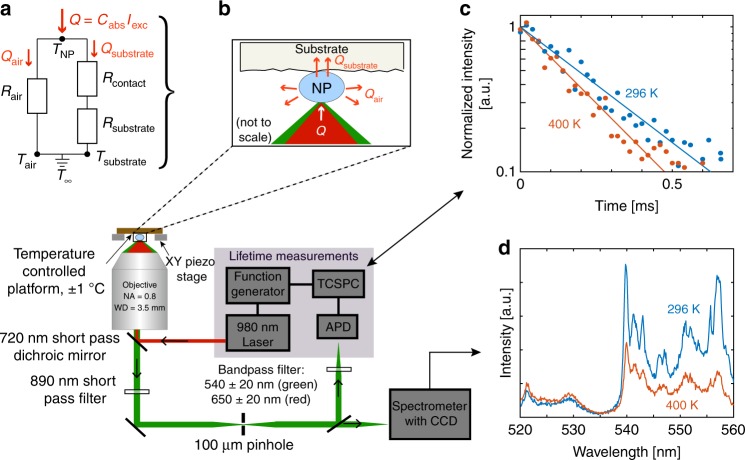
Experimental apparatus and representative raw data. Luminescence intensity ratio and lifetime thermometry measurements of individual nanoparticles dispersed on a substrate. **a** Thermal circuit representation of experiments in which the luminescence intensity ratio or lifetime is measured as a function of *I*_exc_. To indicate that the substrate temperature is allowed to float in these experiments, *T*_substrate_ and *T*_air_ are shorted together and grounded to the environment temperature, *T*_∞_. **b** Schematic of the sample configuration and experimental set-up for both these types of measurements. A spectrometer is used to record single-particle spectra for luminescence intensity ratio measurements. For luminescence lifetime measurements, an avalanche photodiode (APD) and a time-correlated single photon counter (TCSPC) are used. **c** Representative lifetime decay data at two temperatures, fitted with single exponentials. **d** Representative spectra at two temperatures

We test assumptions of negligible self-heating via a series of experiments that systematically probe the components of the thermal circuit shown in Fig. 1a. We employ both faceted hexagonal 50 × 50 × 50 nm^3^ (Mesolight Inc.) and cylindrical 20 × 20 × 40 nm^3^ (synthesized at the Molecular Foundry using standard methods previously described in detail^30^) NaYF_4_ particles doped with 20% Yb^3+^ and 2% Er^3+^. The nanoparticles are excited with a 980 nm laser and imaged using the scanning confocal microscopy set-up^18^ depicted in Fig. 1b. The power absorbed by a single nanoparticle, *Q*, is determined by the intensity of the incident laser beam, *I*_exc_, and the effective nanoparticle absorption cross-section, *C*_abs_. The primary heat dissipation pathways (conduction through the substrate, nanoparticle–substrate contact resistance, and conduction through air) are represented by the three resistors in Fig. 1a. The air-side and substrate-side heat flows are denoted as *Q*_air_ and *Q*_substrate_, respectively, in Fig. 1a, b. In our experiments, we manipulate both *Q* and the thermal resistors.

### Self-heating estimate

We first present a thermal estimate that aims to be highly conservative, i.e. an upper bound on the nanoparticle temperature rise, *θ* = *T*_NP_ − *T*_∞_. Note that for all experiments other than the calibrations, the substrate temperature is allowed to float such that *T*_substrate_ = *T*_air_ = *T*_∞_ (i.e. the environment temperature), as depicted in Fig. 1a. To estimate the temperature rise, we employ the classic concept of thermal resistance, an analogy to electrical resistance applicable for steady-state heat transfer with no internal energy generation^31^. A thermal resistance is defined as the ratio of a temperature difference, *θ*, to the corresponding heat transfer rate, *Q*. This framework has been widely adapted to analyze heat transfer in nanostructures^32,33^ and the analysis we present here can easily be extended to assess laser heating of other types of nanoparticles. The resistors *R*_air_ and *R*_substrate_ relate to three-dimensional heat spreading from the nanoparticle surface into the surrounding air and substrate, respectively, which can be approximated as semi-infinite. We estimate these resistors using conduction shape factors, which are derived from known solutions to the heat conduction equation and tabulated for common geometries^31^. The shape factor, *S*, is defined such that *Q* = *Skθ*, where *k* is the thermal conductivity of the surrounding medium (air or substrate), and the thermal resistance can therefore be expressed as 1/(*Sk*).

As a highly conservative limit we neglect all heat dissipation into the substrate (i.e. *R*_substrate_ + *R*_contact_ ≫ *R*_air_) to consider only the heat lost via conduction through the air surrounding the nanoparticle. Following the thermal circuit in Fig. 1a, the total thermal resistance can be expressed as

1 $$R_{{\mathrm {thermal}}} = \left[ {R_{{\mathrm {air}}}^{ - 1} + \left( {R_{{\mathrm {contact}}} + R_{{\mathrm {substrate}}}} \right)^{ - 1}} \right]^{ - 1}.$$

For *R*_substrate_ + *R*_contact_ ≫ *R*_air_, *R*_thermal_ ≈ *R*_air_ and thus *θ* = *QR*_air_. The incident light is primarily absorbed by the Yb^3+^ ions, which have an absorption cross-section^23,34,35^ of *C*_abs_ ≈ 1 × 10^−20^ cm^2^/ion at 980 nm. For a 20 at.% Yb^3+^ concentration, there are^23^ ~2.8 ions/nm^3^. The upconversion quantum yield of NaYF_4_:Yb^3+^,Er^3+^ nanoparticles has been measured to be very small (<2% in nearly all cases and frequently <0.5%^36–38^). We perform our thermal calculations as though all the absorbed power is converted to heat and thus implicitly assume a quantum yield of zero. This is the most conservative approach, i.e. giving the highest possible modeled temperature rise. Accounting for a finite upconversion quantum yield, or for near-infrared photons from other emission mechanisms such as Er^3+^ downshifting^39^ or downconversion^40^ that are not considered in reported upconversion quantum yield measurements^36–38^, would reduce *Q* and lower our estimated temperature rise, thus increasing the discrepancy between model and experiment.

By approximating the nanoparticle as a half sphere in a semi-infinite medium, we can estimate the thermal resistance as *R*_air_ = 1/(*πk*_air_*D*), where *k*_air_ is the thermal conductivity of air and *D* is the nanoparticle diameter (taken to be 50 nm for the 50 × 50 × 50 nm^3^ particles used in almost all experiments below). To obtain a lower bound on *k*_air_, we take sub-continuum effects into account and reduce the handbook value for *k*_air_ by the factor $D{\mathrm{/}}\left( {2{\mathrm{\Lambda }}} \right)$, where Λ is the mean free path of air (~68 nm at 300 K)^41^. The result for a 50 × 50 × 50 nm^3^ particle is *R*_air_ ≈ 2 × 10^9^ K W^−1^, which is the value we use extensively below for the “conservative model” and is henceforth denoted as *R*_model_. We emphasize that this estimate is conservative because it overpredicts the actual *θ* for a given *Q*, since allowing for finite heat flow through the (*R*_contact_ + *R*_substrate_) branch of the circuit shown in Fig. 1a can only reduce *θ*.

To verify that *R*_model_ ≈ 2 × 10^9^ K W^−1^ is indeed the most conservative estimate, we also considered the possibility of temperature gradients within the nanoparticle and the effects of finite *R*_substrate_ and *R*_contact_ (see Supplementary Note 1 for details). Strictly speaking, the thermal resistance concept does not apply when considering temperature differences within the nanoparticle due to volumetric internal energy generation from the laser excitation. However, we are able to define a quantity with analogous K W^−1^ units (see Supplementary Note 1). The resulting upper bound is *R*_internal_ ~ 2 × 10^6^ K W^−1^, which is negligible compared to *R*_air_ (i.e. Biot number ≪ 1)^31^, and the entire nanoparticle is therefore at a uniform temperature. Meanwhile, allowing for finite *R*_substrate_ (estimated as ~1 × 10^7^ K W^−1^, for a borosilicate glass substrate) and *R*_contact_ (values for nanostructures with similar characteristic lengths^42–44^ on substrates range from 10^4^–10^8^ K W^−1^) can of course only decrease the total thermal resistance further below *R*_air_, which thus remains the most conservative possible value. Yet as we shall shortly see, the large apparent temperature rise observed in experiments would require a thermal resistance over 10 times larger than our upper bound of *R*_model_ ≈ 2 × 10^9^ K W^−1^.

### Ratiometric thermometry calibration and power sweep

**Fig. 2:**
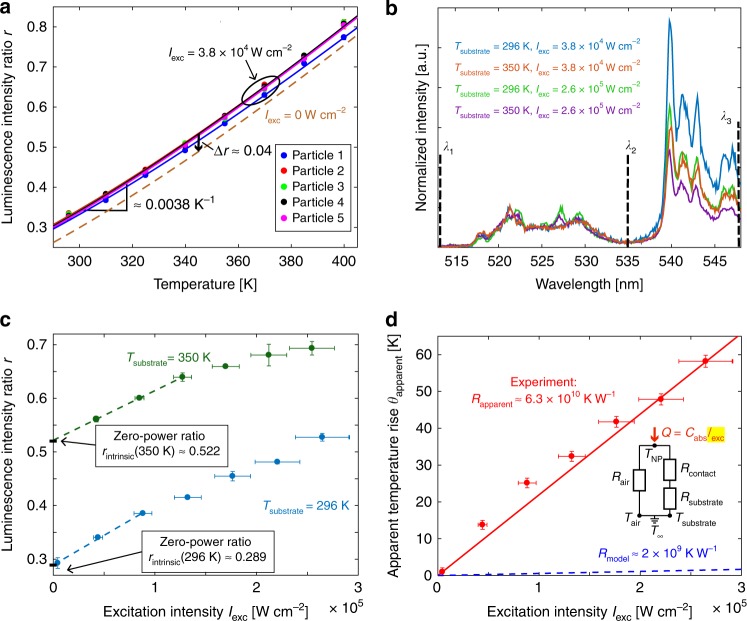
Ratiometric thermometry and apparent self-heating. **a** Luminescence intensity ratio calibration for five individual nanoparticles on a borosilicate glass substrate. The solid lines are fits to Eq. (1). The dashed orange line represents the *r*_intrinsic_(*T*) curve obtained by shifting the best fit *r* (3.8 × 10^4^ W cm^−2^, *T*) curve downward by ∆*r* ≈ 0.04 to account for finite-excitation intensity effects. **b** Luminescence spectra at different *T*_substrate_ and *I*_exc_. The increases in *r* with *T*_substrate_ and *I*_exc_ are both driven by broad changes in the relative emission intensity from *λ*_1_ – *λ*_2_ vs. *λ*_2_ – *λ*_3_. The integration time for all spectra was 60 s. **c** The luminescence intensity ratio increases almost linearly with excitation intensity. The zero-power ratios *r*_intrinsic_(296 K) and *r*_intrinsic_(350 K) can be determined by extrapolating *r*(*I*_exc_, 296 K) and *r*(*I*_exc_, 350 K) to zero power, respectively, here using the bottom three points. Error bars in *r* represent the standard deviation of two consecutive measurements, while error bars in *I*_exc_ are ±10% to account for decreased laser stability and greater measurement uncertainty at higher *I*_exc_. **d** The results from the *T*_substrate_ = 296 K data in **c** converted to an apparent temperature rise using the temperature calibration from **a**. The apparent thermal resistance of ~6.3 × 10^10^ K W^−1^ is directly proportional to the slope of the fitted red line. This value is more than an order of magnitude higher than the 2 × 10^9^ K W^−1^ resistance predicted by our highly conservative thermal model (blue dashed line). Error bars in the apparent temperature rise account for both trial-to-trial variation and uncertainty due to the use of a batch calibration curve (see Supplementary Note 4 for details)

NaYF_4_:Yb^3+^,Er^3+^ is an upconverting system in which the Yb^3+^ sensitizer ions absorb two 980 nm photons and subsequently transfer this energy to a single Er^3+^ ion^23^. The Er^3+^ is excited to its ^4^F_7/2_ state and decays non-radiatively to its ^2^H_11/2_ and ^4^S_3/2_ manifolds^18^. The close spacing of these energy levels gives rise to temperature-dependent luminescence. Radiative relaxation from these manifolds to the ground state results in emission of a single photon in the green wavelength range (~515–565 nm). The temperature-dependent luminescence can be calibrated using^45^

2 $$\frac{{\mathop {\int}\nolimits_{\lambda _1}^{\lambda _2} {I\left( \lambda \right){\mathrm {d}}\lambda } }}{{{\int}_{\lambda _2}^{\lambda _3} {I\left( \lambda \right){\mathrm {d}}\lambda } }} \equiv r = A {\mathrm {exp}}\left( { - \frac{{\Delta E}}{{k_{\mathrm {B}}T}}} \right),$$

where *I*(*λ*) is the emission spectrum, ∆*E* represents the energy difference between ^2^H_11/2_ and ^4^S_3/2_ (theoretical range of 87–100 meV^46^), *k*_B_ is the Boltzmann constant, and *A* is a constant based on the radiative transition rates from ^2^H_11/2_ and ^4^S_3/2_ to ^4^I_15/2_. Thus this temperature-dependent ratio *r* represents the relative emission intensity from ^2^H_11/2_ vs. ^4^S_3/2_. Commonly known as ratiometric thermometry, this self-referenced method is the most popular^2,12^ way to measure temperature using NaYF_4_:Yb^3+^,Er^3+^. In defining *r*, we follow a convention used in some other single-particle thermometry work^17,29^ and choose *λ*_1_ = 513 nm, *λ*_2_ = 535 nm, and *λ*_3_ = 548 nm, which excludes the peak centered at ~556 nm originating from an excited-state to excited-state transition known to occur at high excitation intensities^23,46^. This peak is not captured by the physics governing Eq. (2) and, if included, gives rise to an anomalous power dependence of *r* at the lowest excitation intensities used in this work (see Supplementary Note 3 and Supplementary Fig. 3). Figure 1d shows representative emission spectra at two different temperatures. The sample preparation and single particle identification protocols used here follow those of our previous work^18^ (see Supplementary Note 2 for details). Figure 2a shows the resulting luminescence intensity ratio *r* as a function of temperature for five individual particles. There is excellent uniformity among the five calibration curves, allowing for subsequent temperature measurements via other single particles after this initial batch calibration.

Figure 2b shows that increasing either *T*_substrate_ or *I*_exc_ leads to qualitatively similar changes in *I*(*λ*_1_ − *λ*_2_) relative to *I*(*λ*_2_ − *λ*_3_) that increase *r*. For example, the spectrum obtained for *T*_substrate_ = 350 K with *I*_exc_ = 3.8 × 10^4^ W cm^−2^ is nearly identical to that for *T*_substrate_ = 296 K with an increased *I*_exc_ = 2.6 × 10^5^ W cm^−2^. Further increasing *T*_substrate_ to 350 K while holding *I*_exc_ at the higher value of 2.6 × 10^5^ W cm^−2^ causes an additional relative intensity change and *r* again increases. Figure 2c shows a broader range of the *r*(*I*_exc_) response for both fixed *T*_substrate_ = 296 K and *T*_substrate_ = 350 K. The most direct interpretation of the increase in *r* is that the particle is heating up by an amount *θ*_apparent_ = *C*_abs_*I*_exc_*R*_apparent_, where *R*_apparent_ is the apparent thermal resistance between the particle and substrate. Results such as Fig. 2c can thus be used to evaluate *R*_apparent_ experimentally for comparison with the *R*_model_ estimate given previously. To facilitate this comparison, we linearize the *r*(*I*_exc_,*T*) response function to write *r*(*I*_exc_, *T*) = *r*_intrinsic_(*T*) + *α*(*T*)*C*_abs_*I*_exc_*R*_apparent_, where $\alpha \left( T \right) = \left[ {\frac{{\partial r}}{{\partial T}}} \right]_{I_{{\mathrm {exc}}}}$ is the local slope of the *r*(*T*) calibration curve at some temperature *T*, e.g. *α* ≈ 0.0038 K^−1^ near 300 K in Fig. 2a, and *r*_intrinsic_(*T*) is the intrinsic calibration curve in the absence of any self-heating artifacts. This approach is analogous to the manner in which self-heating effects are quantified in electrical resistance thermometry, where the finite measurement current leads to Joule heating of the probe; here, the zero-power ratio *r*_intrinsic_(*T*) is analogous to concept of cold-wire resistance^47^.

To minimize finite-excitation intensity artifacts in the calibration, we use a relatively low intensity of *I*_exc_ = 3.8 × 10^4^ W cm^−2^ for our *r*(*T*) calibration in Fig. 2a. The most rigorous way to obtain an intrinsic *r*(*T*) calibration curve would be to measure *r*(*I*_exc_,*T*) at several intensities for each substrate temperature *T*, and extrapolate each curve to find $r_{{\mathrm {intrinsic}}}\left( T \right) = \mathop {{\lim }}\nolimits_{I_{{\mathrm {exc}} \to 0}} \left( {r\left( {I_{{\mathrm {exc}}},T} \right)} \right)$The dashed blue and green lines in Fig. 2c show such an extrapolation at *T*_substrate_ = 296 K and *T*_substrate_ = 350 K, respectively. At very low *I*_exc_, the error induced by excitation intensity artifacts is minimal. For example, Fig. 2c shows that the extrapolated *r*_intrinsic_(296 K) = 0.289, while the lowest measured point used a finite *I*_exc_ = 4.4 × 10^3^ W cm^−2^, at which *r*(4.4 × 10^3^ W cm^−2^, 296 K) = 0.293, a small increase of Δ*r* = 0.004 above *r*_intrinsic_. Because *α* ≈ 0.0038 K^−1^ near room temperature, this Δ*r* corresponds to an apparent temperature shift of *θ* = Δ*r*/α = 1.1 K, negligible for most purposes. However, at the highest-*I*_exc_ point, *r*(2.6 × 10^5^ W cm^−2^, 296 K) = 0.527, with Δ*r* = 0.238 corresponding to a much larger temperature error of ~60 K. To compensate for finite-excitation intensity artifacts in the calibration, we generate *r*_intrinsic_(*T*) by shifting the best fit *r*(3.8 × 10^4^ W cm^−2^, *T*) curve of Fig. 2a downward by a uniform ∆*r* ≈ 0.04, which corresponds to a temperature shift of 8 K using the *α* ≈ 0.0038 K^−1^ slope. This *r*_intrinsic_(*T*) curve is shown as the orange dashed line in Fig. 2a. Although as noted previously the most rigorous construction of an *r*_intrinsic_(*T*) curve would require measuring *r*(*I*_exc_,*T*) at multiple *I*_exc_ for each *T*_substrate_, extrapolation of the data in Fig. 2c gives ∆*r* ≈ 0.04 for both *T*_substrate_ = 296 K and *T*_substrate_ = 350 K, and this uniform shift is thus a reasonable approximation over the 296–400 K temperature range used in this work. As a consequence of this approximation, the extrapolated *I*_exc_ = 0 W cm^−2^ (i.e. *r*_intrinsic_(*T*)) curve has the same local slope at each *T* as the best fit *r*(3.8 × 10^4^ W cm^−2^, *T*) curve.

These artifacts drive one key practical takeaway of this work: there is an inherent trade-off between minimizing artifacts in the ratiometric thermometry signal and maximizing signal to noise. While conventional wisdom suggests that high excitation intensities should be used to increase the emission intensity (until optical saturation is reached), our results show that ratiometric temperature measurements should be performed at the lowest excitation intensity that provides sufficient signal to noise. Alternatively, both the calibration and all subsequent measurements can be carried out at a single *I*_exc_ of any magnitude, but this approach places far more stringent demands on the laser stability. We note that these two strategies can be applied even in scenarios where the *I*_exc_ exciting a nanoparticle cannot be measured, e.g. for nanoparticles deposited in absorbing and scattering biological media. If both the biological sample and the depth at which the nanoparticles are deposited are fairly uniform, the latter strategy of maintaining a constant *I*_exc_ simply requires monitoring the laser power at some location in the optical path; otherwise, a particle-by-particle calibration is required.

Figure 2d shows the *T*_substrate_ = 296 K ratio data in Fig. 2c expressed as an apparent temperature rise vs. excitation intensity. The slope of the best-fit line implies an apparent thermal resistance of *R*_apparent_ ≈ 6.3 × 10^10^ K W^−1^, more than an order of magnitude higher than the highly conservative bound of *R*_model_ ≈ 2 × 10^9^ K W^−1^ presented above. This dramatic discrepancy motivates all subsequent investigation in this work because it indicates that either some model input parameters we presumed to be well-known deviate dramatically from their expected values, or else the effects we measure are fundamentally non-thermal in nature, the latter of which we will show to be the case.

### Luminescence lifetime thermometry and power sweep

**Fig. 3:**
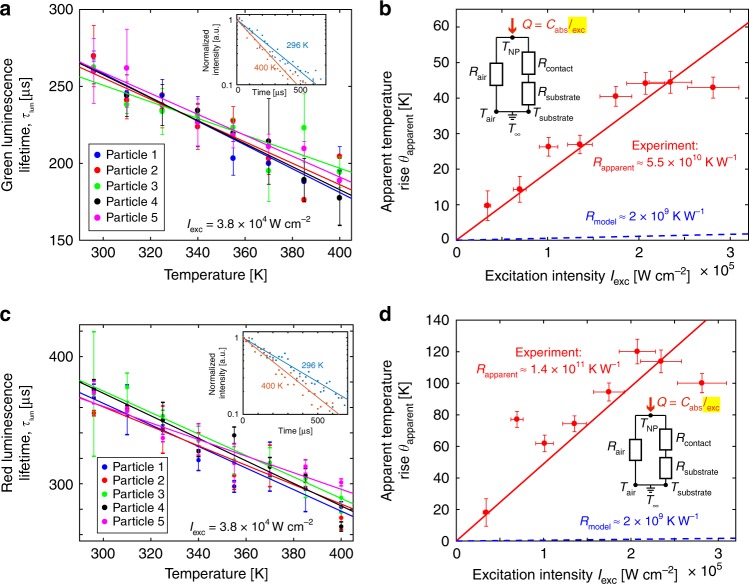
Luminescence lifetime thermometry and apparent self-heating. **a** Temperature-dependent luminescence lifetime calibration for the green emission band (540 ± 20 nm) using five individual nanoparticles on a borosilicate glass substrate, along with linear fits. The excitation modulation for all measurements was a 50 Hz square wave (50% duty cycle), and the total collection time was 30 s. Error bars in *τ*_lum_ represent the standard deviation of two consecutive measurements, while error bars in *I*_exc_ are ±10% to account for decreased laser stability and greater measurement uncertainty at higher *I*_exc_. **b** Apparent temperature rise as a function of excitation intensity measured using the luminescence lifetime of the green spectral band for a single nanoparticle. The substrate temperature was allowed to float such that *T*_substrate_ = *T*_air_ = 296 K. The experimentally measured apparent thermal resistance is ~5.5 × 10^10^ K W^−1^, in remarkably good agreement with the value of 6.3 × 10^10^ K W^−1^ obtained from ratiometric measurements, and more than an order of magnitude higher than the conservative model prediction of 2 × 10^9^ K W^−1^. Error bars in the apparent temperature rise account for trial-to-trial variation and uncertainty due to the use of a batch calibration curve (see Supplementary Note 5 for details). **c**, **d** Similar results for the red emission band (650 ± 20 nm) using the same five nanoparticles. The apparent thermal resistance from the red band is within a factor of 2.5 of that from the green band

To further investigate whether this change in the luminescence ratio with excitation intensity could be thermal, we employed a complementary technique based on the lifetime of the luminescence emitted by a nanoparticle. Though less common than the ratiometric method, this lifetime technique has been used previously at the ensemble level^48,49^. Here, we present the first demonstration using a single UCNP. Lifetime decay curves were obtained using a time-correlated single photon counter (TCSPC) to tag the arrival times of collected photons with respect to the modulated output of the 980 nm excitation laser. We measure the combined lifetime of the ^2^H_11/2_ and ^4^S_3/2_ excited states, which are assumed to be in thermal equilibrium, and fit the time-resolved luminescence data to an exponential decay. Figure 3a shows the lifetime (*τ*_lum_) vs. temperature calibration curve for the green emission band using five individual nanoparticles. We measure lifetimes of hundreds of microseconds, consistent with other single-particle measurements^23^. The luminescence lifetime clearly decreases with increasing temperature, likely due to an increase in phonon-mediated decay^48^. Although more sophisticated models to capture the temperature dependence of the non-radiative component of the luminescence lifetime exist^50^, we follow the empirical approach of Savchuk et al.^48^ and fit the temperature-dependent lifetime data to a linear model in Fig. 3a, which is a suitable approximation over this temperature range. The particle-to-particle uniformity and repeatability from one trial to another are sufficiently consistent to allow for batch-calibrated temperature measurements.

Having demonstrated the validity of luminescence lifetime thermometry, we measured the apparent temperature rise as a function of excitation intensity using this method, analogous to the ratiometric power sweep shown in Fig. 2d. Figure 3b shows the results based on the lifetime of the green emission band. We measure an apparent temperature rise that is quantitatively consistent with the ratiometric method. Due to the modulated laser excitation (50 Hz square wave, 50% duty cycle), we must consider the thermal time constant of the nanoparticle relative to its luminescence lifetime in order to assess the validity of the thermal resistance framework, which is applicable only for steady-state heat transfer. The thermal time constant can be expressed as

3 $$\tau _{{\mathrm {thermal}}} = \rho cV \cdot R_{{\mathrm {thermal}}},$$

where *ρ*, *c*, and *V* are the density, heat capacity, and volume of the nanoparticle, respectively. We use *ρc* ≈ 3 × 10^6^ J m^−3^ K^−1^, a nearly constant value for all fully dense solids above their Debye temperatures^51^, which is expected to be the case for NaYF_4_ at room temperature based on data for related materials^52^. If we take *R*_thermal_ to be *R*_model_ ≈ 2 × 10^9^ K W^−1^, *τ*_thermal_ ≈ 1 μs. If we instead use the value of 6.3 × 10^10^ K W^−1^ measured via the ratiometric method, *τ*_thermal_ ≈ 20 μs. In either case, *τ*_thermal_ is at least an order of magnitude smaller than the measured *τ*_lum_ values, so the system can be considered quasi-DC from a thermal standpoint and the thermal resistance framework thus remains valid.

The apparent thermal resistance we measure using the luminescence lifetime method is ~5.5 × 10^10^ K W^−1^, which agrees remarkably well with the value of 6.3 × 10^10^ K W^−1^ obtained from ratiometric measurements. Here, we note that excitation intensity dependence of the luminescence lifetime has been reported previously for individual NaYF_4_:Yb^3+^,Er^3+^ nanoparticles. Participation of the green emitting states in energy transfer processes with other excited states, rather than elevated temperature, has been proposed to explain these effects^23^. Nonetheless, the striking consistency of apparent *R* values estimated from our luminescence lifetime and ratiometric measurements could be construed as supporting a thermal interpretation. Furthermore, we measure the lifetime of the red emission and again observe a large apparent temperature rise. Figure 3c shows the temperature calibration for the red emission band using the same nanoparticles as Fig. 3a, d shows the apparent temperature rise measured with the same nanoparticle as Fig. 3c. The apparent thermal resistance is ~1.4 × 10^11^ K W^−1^, about a factor of 2.5 larger than the analogous values obtained from the ratiometric and green emission lifetime approaches. While the physical origins of the temperature-dependent luminescence lifetime of the green spectral band and the spectral shifts in this same band used for ratiometric measurements may not be completely independent, with the red emission there is no ambiguity: we measure the lifetime of the ^4^F_9/2_ excited state, distinct from the ^2^H_11/2_ and ^4^S_3/2_ excited states corresponding to the green emission. Yet, despite using a temperature metric based on a completely different spectral band, we again measure an apparent thermal resistance over an order of magnitude larger than *R*_model._

### Manipulating the thermal resistors and *C*_abs_

While both the ratiometric and luminescence lifetime power sweeps indicate a dramatic apparent temperature rise, over an order of magnitude higher than conservative thermal models predict, these experiments rely solely on varying the excitation intensity. Increasing *I*_exc_ affects not only the temperature rise, but also the population of excited states and the rates of certain energy transfer processes. Thus in order to more directly assess whether or not the results were thermal in nature (i.e. indicative of a true temperature rise), we tested the effects of modifying *R*_substrate_, *R*_contact_, and *C*_abs_.

**Fig. 4:**
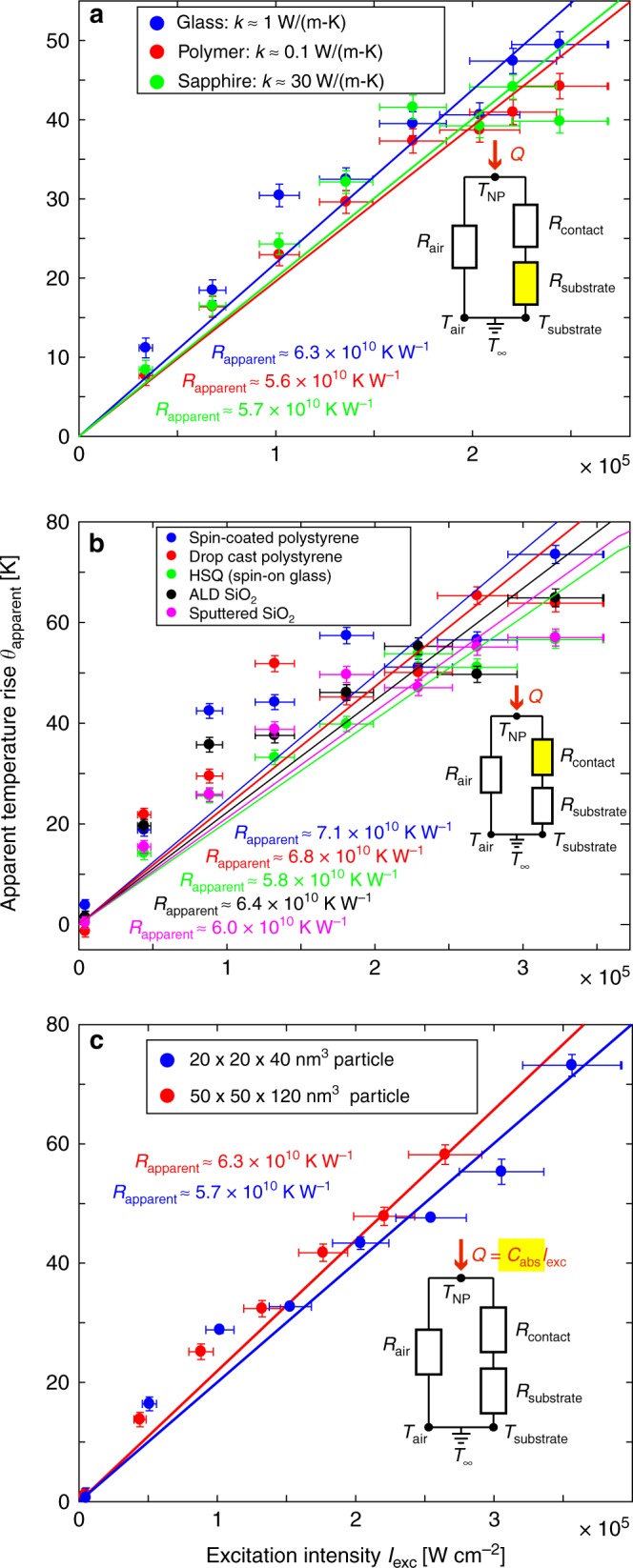
Manipulating the thermal environment. Apparent temperature rise as a function of excitation intensity measured using the ratiometric method for various manipulations of the thermal environment. The modified component of the thermal circuit in each case is highlighted in yellow. Error bars in the apparent temperature rise account for both trial-to-trial variation and uncertainty due to the use of a batch calibration curve (see Supplementary Note 4 for details), while error bars in *I*_exc_ are ±10% to account for decreased laser stability and greater measurement uncertainty at higher *I*_exc_. **a** *R*_substrate_ is modified by using three different substrates with thermal conductivities spanning over two orders of magnitude. **b** *R*_contact_ is modified using five different coating protocols. **c** *C*_abs_ is varied by using nanoparticles of two different sizes, a 20 × 20 × 40 nm^3^ particle and a 50 × 50 × 50 nm^3^ particle (the latter repeated from Fig. 2b), both on borosilicate glass substrates. Despite all these manipulations of the thermal environment, the apparent thermal resistance remains similar to the baseline ratiometric value of 6.3 × 10^10^ K W^−1^. **a**, **b** used 50 × 50 × 50 nm^3^ particles, and **a**, **c** were uncoated

First, we varied *R*_substrate_ by repeating the experiment on three different IR-transparent substrates with thermal conductivities spanning over two orders of magnitude (*k*_polymer_ ≈ 0.1 W m^−1^ K^−1^ (polyester), *k*_glass_ ≈ 1 W m^−1^ K^−1^, and *k*_sapphire_ ≈ 30 W m^−1^ K^−1^) and again measuring the apparent temperature rise via the ratiometric method as a function of *I*_exc_. As shown in Fig. 4a, the apparent temperature rise is essentially identical for all three substrates. This result clearly indicates that if the artifact is thermal, it is insensitive to *R*_substrate_, which means one of the following must be true: (i) most heat flows into the air, and thus (*R*_substrate_ + *R*_contact_) ≫ *R*_air_; (ii) most heat flows into the substrate, subject to *R*_air_ ≫  *R*_contact_ ≫ *R*_substrate_; or (iii) both pathways are important, with *R*_air_ ~ *R*_contact_ ≫ *R*_substrate_. However, we note that our previous estimate for *R*_air_ (i.e. *R*_model_ ≈ 2 × 10^9^ K W^−1^) is over two orders of magnitude larger than our estimate of *R*_substrate_ ≈ 1 × 10^7^ K W^−1^, suggesting that case (ii) is the most likely among these three options. Here, Eq. (1) simplifies to *R*_thermal_ ≈ *R*_contact_, and thus *θ* = *Q*_abs_*R*_contact_.

Next, to test the possibility of *R*_contact_ being the limiting thermal resistance, we applied five different coating protocols to our nanoparticle samples, designed to improve the thermal coupling between the particle and substrate and thus reduce *R*_contact_. If case (ii) from the previous paragraph is correct, these coatings should result in substantial reductions in the observed *θ*. Nanoparticles were spin coated on borosilicate glass substrates using our normal sample preparation protocol and the following coatings were applied subsequently: drop cast and spin-coated polystyrene (~1 μm thick), hydrogen silsesquioxane (HSQ) spin-on glass (~50 nm), sputtered SiO_2_ (~100 nm), and atomic layer deposition (ALD) of SiO_2_ (~50 nm). ALD in particular ensures highly conformal deposition, even over nanoscale features with high aspect ratios, due to the use of gas phase precursors.

Figure 4b shows the results of measuring the apparent temperature rise *θ*_apparent_ using the ratiometric method for these five different coatings. In all cases we find a response between 5.8–7.1 × 10^10^ K W^−1^, very similar to the artifact observed in the uncoated samples. Here, we note that changing *R*_contact_ will change *R*_air_ as well. The coating places a new resistor in series between the particle and air, but also acts as a heat spreader, providing more surface area and thus reducing *R*_air_. While these are competing effects that could in principle cancel out, we consider it extremely unlikely that such cancellation could occur in all five cases, considering the diversity of coating protocols, materials, and thicknesses. Rather, the nearly constant slope seen in Fig. 4b for all five coatings indicates that *R*_contact_ does not limit the heat dissipation; furthermore, it indicates that *R*_air_ is not the limiter either because the effective *R*_air_ also changed due to the series resistance and heat spreading effects just mentioned. Thus, the combined results of Fig. 4a, b imply that none of the three external thermal resistors controls the apparent temperature rise.

The final parameter we vary is the effective absorption cross-section of the nanoparticle, *C*_abs_. To keep the electron energy transfer pathways as constant as possible, we hold the dopant concentrations constant and manipulate *C*_abs_ by changing the nanoparticle size (and thus the total number of absorbing Yb^3+^ ions). Thus *C*_abs,_ and therefore *Q*, will scale as *D*^3^. Figure 4c shows the apparent temperature rise for two different nanoparticle sizes (50 × 50 × 50 nm^3^ and 20 × 20 × 40 nm^3^, the latter being the same particles used in our previous work^18^). The apparent temperature rise is quite consistent between the two particle sizes. For the temperature rise to be independent of particle size, the thermal resistance would have to scale as 1/*D*^3^. However, referring to our previous calculations, none of the external thermal resistors in our thermal circuit can give a stronger inverse power law scaling than 1/*D*^2^, which corresponds to the case of *R*_air_ ~ 1/(*k*_air_*D*) ~ 1/*D*^2^ if sub-continuum effects are considered and thus *k*_air_ ~ *D*. Consequently, this result corroborates our conclusion from manipulating the resistors: the apparent temperature rise we observe cannot actually be self-heating limited by external thermal resistances.

### Rate equation modeling of *r* vs. *I*_exc_

**Fig. 5:**
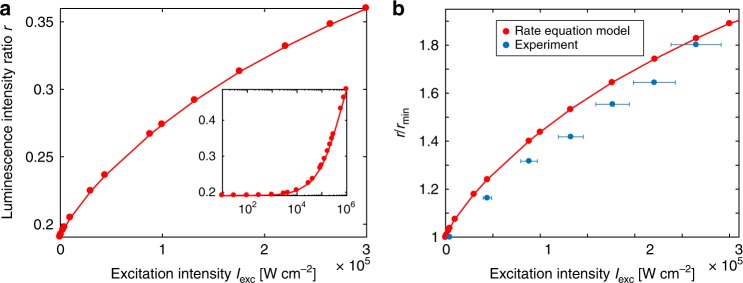
Rate equation modeling. **a** Modeled *r*(*I*_exc_) using rate equation analysis at a constant temperature of *T* *=* 298 K. The main plot shows the results over the excitation intensity range used in the majority of our experiments. The inset shows the modeled *r*(*I*_exc_) over a larger *I*_exc_ range. **b** Comparison of the measured (Fig. 2c, 296 K) and modeled relative increase in *r* with *I*_exc_. Both the modeled and measured *r*(*I*_exc_) show a similar percent increase, further confirming that this effect is non-thermal in nature and can largely be explained by radiative and non-radiative relaxation from higher-lying Er^3+^ energy levels that become increasingly populated at higher *I*_exc_. Error bars in *I*_exc_ for the experimental data are ±10% to account for decreased laser stability and greater measurement uncertainty at higher *I*_exc_

Having demonstrated that the apparent self-heating artifact cannot indicate a true temperature rise, we instead hypothesized that energy relaxation from higher-lying Er^3+^ energy levels can distort the Boltzmann population distribution of the ^2^H_11/2_ and ^4^S_3/2_ excited states and increase *r*. In order to test this hypothesis, we employed rate equation analysis developed in our previous work^53,54^ using a framework based on Judd–Ofelt theory^55^ and energy transfer models from Kushida^56^. Steady-state solutions to these rate equation models indeed predict an increase in *r* with excitation intensity comparable in magnitude to our experimental results, using a constant modeled temperature of *T* = 298 K and without the addition of any ad hoc intensity-dependent parameters. Figure 5a shows that the modeled luminescence intensity ratio increases by ~75% over the excitation intensity range used in the majority of our experiments, similar to the ~80% increase observed experimentally. The modeled *r* values are slightly lower than the experimental *r* values; however, the relative change in *r* is more scientifically relevant than the absolute values since they are highly dependent on the semi-empirical parameters employed in the idealized rate equations. When the *r*(*I*_exc_) data are normalized to their respective minimum *r* values, as in Fig. 5b, the agreement between the modeled and measured trends is clearly evident.

The steady-state increase in the modeled *r* values can be explained by the fact that non-linear effects become more prominent at high-excitation intensities, resulting in a shift of stored energy to higher Er^3+^ levels, particularly after saturation of lower levels (see Supplementary Figure 4). This increased population of higher-lying Er^3+^ levels drives two main phenomena that give rise to the excitation intensity dependence of *r*. First, these highly excited Er^3+^ states emit additional green photons that preferentially increase the integrated emission intensity of the *λ*_1_ − *λ*_2_ wavelength band relative to the *λ*_2_ − *λ*_3_ band, thus increasing *r*. In other words, if these additional green photons add a similar number of counts to each band, the percent increase in the integrated intensity of the *λ*_1_ − *λ*_2_ band will be larger than that of the *λ*_2_ − *λ*_3_ band; consequently, *r* will increase. While many of these additional transitions are closely spaced and of sufficient breadth such that they manifest as broadband intensity changes, certain transitions can be observed as distinct features in the experimental high-excitation intensity spectra shown in Fig. 2b. For example, a new peak appears at ~527 nm, which we attribute to Er^3+ 2^P_3/2_ to ^4^I_9/2_ radiative relaxation.

A second mechanism contributing to the increase in *r* is that relaxation of ions populated in energy levels above the ^2^H_11/2_ and ^4^S_3/2_ manifolds results in appreciable energy flowing into the higher ^2^H_11/2_ manifold from above, thereby increasing the intensity ratio between the ^2^H_11/2_ and ^4^S_3/2_ populations (see Supplementary Note 6 and Supplementary Fig. 4). At low-excitation intensities, multiphonon relaxation from the ^2^H_11/2_ level to the ^4^S_3/2_ level is sufficiently rapid to thermally equilibrate the levels, minimizing this phenomenon. However, at higher excitation intensities, non-radiative relaxation to the ^2^H_11/2_ manifold from highly populated, higher energy manifolds becomes sufficiently rapid such that it can imbalance this thermal equilibrium. Thus, these modeling results provide a physical mechanism for the increase in *r* with *I*_exc_ that we observe experimentally and further confirm that this effect is non-thermal in nature. This type of rate equation analysis could in principle be extended to other UCNP compositions of interest for thermometry.

### Frequency-dependent apparent temperature rise

In the previous sections, we focused on continuous wave and low-frequency (50 Hz square wave, which is quasi-DC for this system) measurements. We now consider the effects of modulated excitation at much higher frequencies on the ratiometric thermometry method, which is important for two reasons. First, modulated excitation is common in UCNP experiments such as luminescence lifetime measurements and has been proposed for other purposes, such as mitigating thermal loading during optical trapping^17,29^, reducing tissue damage in biological applications^57^, and manipulating the color of emitted light^58^. Second, the frequency response to periodic heating gives rise to characteristic behavior from which thermal resistance can often be determined^33^ without knowing the absorbed power, and thus in principle provides a powerful alternative to the methods above for determining *R*_apparent_. Here we develop a combined thermal and luminescence model to explain the measurement response, which compares well with additional experiments presented below. From the accessible experimental regime we can only extract an upper bound on *R*_apparent_ ≈ 5 × 10^11^ K W^−1^, which is consistent with both the empirically observed and conservatively modeled resistance values presented above.

We model the nanoparticle as a cylinder with a spatially uniform (lumped) temperature profile and account for heat loss to the environment via a generic thermal resistor,

4 $$Q\left( t \right) - \frac{{\theta (t)}}{{R_{{\mathrm {thermal}}}}} = \rho cV\frac{{{\mathrm {d}}\theta (t)}}{{{\mathrm {d}}t}},$$

where *Q*(*t*) is a square wave oscillating between 0 and *Q*_max_ with a 50% duty cycle that represents the power absorbed by the nanoparticle from the modulated excitation laser, *θ*(*t*) = *T*(*t*) *−* *T*_∞_ where *T*(*t*) is the nanoparticle temperature as a function of time and *T*_∞_ is the temperature of the surroundings. The temperature rise measured via the ratiometric method differs from more conventional thermometry methods in two key ways. First, the measured average of a time-varying temperature rise is weighted by the corresponding time-varying luminescence intensity, since the average spectrum recorded by the spectrometer is weighted by the counts it receives. Thus, because higher excitation intensities result in more emitted photons (until optical saturation is reached^18^), the temperature rise associated with these higher intensities is disproportionately represented in the measured average. Conversely, the temperature rise associated with lower excitation intensities is weighted less heavily; indeed, when the nanoparticle is not emitting at all, no temperature information is contributed to the final average. The second difference is that the luminescence lifetime of the nanoparticle introduces an additional timescale to the experiment. Mathematically, we capture these features by expressing the measured temperature rise as a photon flux-weighted average of the true temperature rise, as follows:

5 $$\theta _{{\mathrm {measured}}}\left( {f_{{\mathrm {exc}}}} \right) = \frac{{\mathop {\int}\nolimits_0^{\frac{1}{{f_{{\mathrm {exc}}}}}} {I_{{\mathrm {lum}}}\left( t \right)\theta \left( t \right){\mathrm {d}}t} }}{{\mathop {\int}\nolimits_0^{\frac{1}{{f_{{\mathrm {exc}}}}}} {I_{{\mathrm {lum}}}\left( t \right){\mathrm {d}}t} }},$$

where *f*_exc_ is the excitation frequency and *I*_lum_ is the luminescence intensity. In our experiments we ensure that we have reached the steady-periodic regime of *I*_lum_(*t*) before defining *t* = 0 in Eq. (5); additionally, the spectrometer integration time (60 s) is much larger than the other relevant timescales. Certain second-order effects, such as the temperature dependence of the luminescence lifetime and the luminescence intensity, are neglected in this formulation (see Supplementary Note 7).

**Fig. 6:**
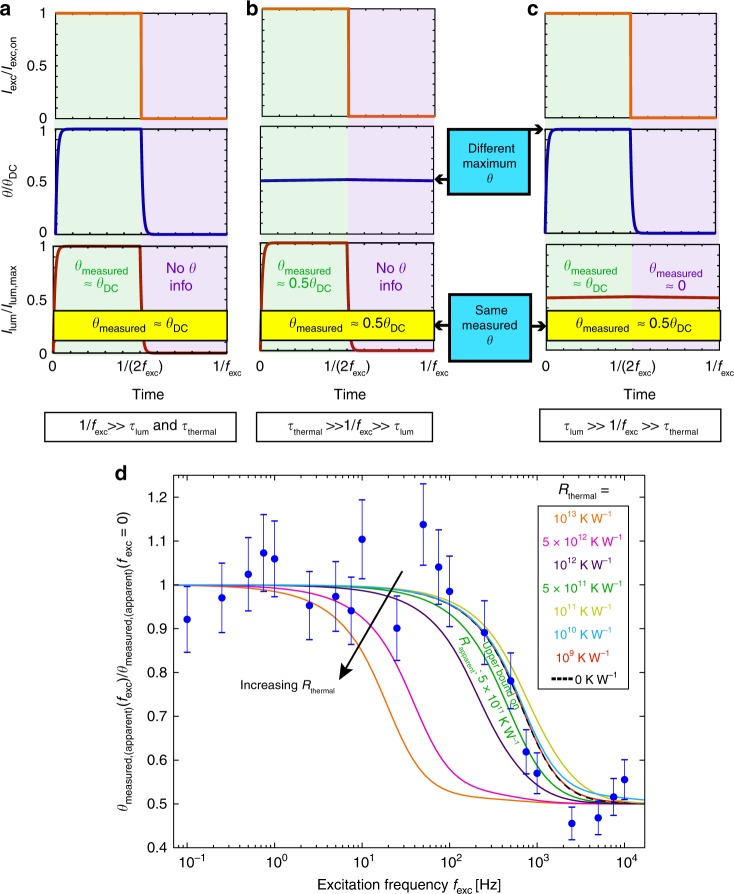
Frequency-dependent ratiometric measurements. Complications when interpreting ratiometric thermometry results obtained with periodic laser excitation at higher frequencies. **a** When 1/*f*_exc_  ≫ *τ*_lum_ and *τ*_thermal_, the temperature rise, *θ*, is a square wave between *0* and *θ*_DC_. Similarly, the luminescence intensity, *I*_lum_, is a square wave between 0 and *I*_lum,max_. Thus *θ*_measured_ = *θ*_DC_ is an average over the half cycle when *I*_exc_ is on. **b** When *τ*_thermal_ ≫ 1/*f*_exc_ ≫ *τ*_lum_, the steady-periodic temperature rise is nearly constant at 0.5*θ*_DC_*. θ*_measured_ is again an average over the half cycle when *I*_exc_ is on, and thus here *θ*_measured_ = 0.5*θ*_DC_. **c** If instead *τ*_lum_ ≫ 1/*f*_exc_ ≫ *τ*_thermal_, *θ* is a square wave between *0* and *θ*_DC_, but now *I*_lum_ is nearly constant at 0.5*I*_lum,max_. Now, *θ*_measured_ represents an average over the full cycle. Thus once again *θ*_measured_ = 0.5*θ*_DC_, but for a completely different reason than the scenario in **b**. The inability to distinguish between these two cases leads to a loss in sensitivity to *R*_thermal_ if *τ*_lum_ *>* *τ*_thermal_. **d** Modeled temperature rise (lines) and experimental apparent temperature rise (points) as a function of excitation frequency for a single 50 × 50 × 50 nm^3^ nanoparticle on an uncoated borosilicate glass substrate. A function generator modulates the output of the 980 nm laser diode as a square wave up to ~10 kHz (see Supplementary Note 8 and Supplementary Fig. 5). Fitting the experimental points yields an upper bound *R*_apparent_ of 5 × 10^11^ K W^−1^, which is consistent with both the estimated values of ~ 6 × 10^10^–1 × 10^11^ K W^−1^ from other experiments and the value of 2 × 10^9^ K W^−1^ predicted by our conservative thermal model, though cannot help distinguish between them. Error bars in the experimental data account for both trial-to-trial variation and the normalization by *θ*_measured,apparent_*(f*_exc_ = 0) (see Supplementary Note 9 for details)

At lower frequencies such that 1/*f*_exc_ ≫ *τ*_lum_ and *τ*_thermal_, *θ*(*t*) is approximately a square wave between 0 and some peak value *θ*_DC_, and similarly *I*_lum_(*t*) is approximately a square wave between 0 and some peak *I*_lum,max_. Both *θ*(*t*) and *I*_lum_(*t*) are synchronized with the *I*_exc_(*t*) square wave. *θ*_DC_ is defined as the steady-state *θ* expected from a constant heating *Q* = *Q*_max_ = *C*_abs_*I*_exc,on_. Therefore *θ*_measured_ is an average of *θ*(*t*), but averaged only over the half cycle when *I*_exc_ is on; here, *θ*_measured_ = *θ*_DC_ (Fig. 6a).

The most significant consequences of Eq. (5) occur at higher frequencies. If *τ*_thermal_  ≫ 1/*f*_exc_ ≫ *τ*_lum_, the temperature cannot keep up with the laser heating oscillations and *θ*(*t*) → 0.5*θ*_DC_. *I*_lum_(*t*) is still a square wave and *θ*_measured_ is thus an average of *θ*(*t*) over the half cycle when *I*_exc_ is on, so *θ*_measured_ = 0.5*θ*_DC_ (Fig. 6b). Therefore, for *τ*_thermal_ ≫ *τ*_lum_, the frequency at which *θ*_measured_ transitions from *θ*_DC_ to 0.5*θ*_DC_ corresponds to *f*_exc_*τ*_thermal_ ~ 1, allowing *τ*_thermal_ to be determined experimentally and thus *R*_thermal_ from the full solution to Eq. (5), all without knowing *C*_abs_.

The frequency regime that can lead to erroneous conclusions is *τ*_lum_ ≫ 1/*f*_exc_ ≫ *τ*_thermal_ (Fig. 6c). Here, *θ*(*t*) remains a square wave between 0 and *θ*_DC_, but now the luminescence cannot keep up with the *I*_exc_ oscillations and *I*_lum_(*t*) → 0.5*I*_lum,max_, such that luminescent photons are emitted continuously over the full cycle. Thus, *θ*_measured_ now represents an average of *θ*(*t*) over the full cycle. Consequently, even though the true *θ*(*t*) still reaches *θ*_DC_, the spectral ratio will correspond to 0.5*θ*_DC_, but for a reason that has nothing to do with *f*_exc_*τ*_thermal_ ~ 1 (Fig. 6c). The crucial conclusion is that there are two completely different mechanisms that can cause a transition from *θ*_measured_ = *θ*_DC_ → 0.5*θ*_DC_ with increasing *f*_exc_. The inability to distinguish between these two scenarios results in a loss of sensitivity to *R*_thermal_ when *τ*_lum_ ≫ *τ*_thermal_, which unfortunately thermal estimates suggest is the regime of our experiments.

We obtain an analytical solution for *θ*_measured_ (see Supplementary Note 7) and plot this calculated temperature rise as a function of frequency for several values of *R*_thermal_ in Fig. 6c along with the experimental results for *θ*_measured, apparent_. By comparing the experimental points and modeled curves, we conclude that our experiments operate in the regime that loses sensitivity to *R*_thermal_. Consequently, we are only able place an upper bound on *R*_apparent_ of ~ 5 × 10^11^ K W^−1^. While this upper bound is consistent with the estimated values of ~6 × 10^10^–1 × 10^11^ K W^−1^ from all continuous wave and low-frequency experiments, it is also consistent with the value of 2 × 10^9^ K W^−1^ predicted by our conservative thermal model. These results imply that caution must be taken when interpreting ratiometric thermometry results obtained using modulated excitation.

### Extension to other particle compositions and environments

In this work, we have emphasized the configuration of a single NaYF_4_:Yb^3+^,Er^3+^ nanoparticle surrounded by air. We also performed additional experiments that show many of our key findings can be generalized to other common application scenarios. For example, we measured *r*(*I*_exc_) for a nanoparticle surrounded by a drop of deionized water rather than air (Supplementary Note 10 and Supplementary Fig. 6). The measured *r* values are similar whether the nanoparticle is surrounded by air or water, despite the fact that water has a thermal conductivity an order of magnitude higher than that of air. This result further strengthens the conclusion of Fig. 4 that none of the external thermal resistors controls the apparent temperature rise, and moreover extends the validity of our results to aqueous media, which is relevant for biological applications of UCNPs^59^. We also observe a similar apparent self-heating artifact for clusters of two 50 × 50 × 50 nm^3^ particles (Supplementary Note 11 and Supplementary Fig. 7), despite changes to the heat dissipation pathways in this scenario, again corroborating our conclusion that the apparent self-heating artifact is non-thermal and demonstrating that the artifact extends to small nanoparticle ensembles. Figure 4c shows that this artifact is quantitatively similar (i.e. *R*_apparent_ is the same within 6%), for 20 × 20 × 40 and 50 × 50 × 50 nm^3^ nanoparticles synthesized by two different groups, suggesting that this value of *R*_apparent_ is a broadly applicable metric for NaYF_4_ nanoparticles doped with 20% Yb^3+^ and 2% Er^3+^. Indeed, we expect that qualitatively similar *r*(*I*_exc_) behavior is likely for other UCNP compositions, particularly in the case of Yb^3+^/Er^3+^ co-doping of host matrices with similar maximum phonon energies. Our experiments used NaYF_4_ because it is the most common UCNP host material^5,12,60^. We note that the most important host matrix property affecting the upconversion process is its maximum phonon energy^54,59^, which is ~350 cm^−1^ for NaYF_4_. We have reviewed a selection of other typical host materials, including various fluorides (e.g. NaLuF_4_, NaNdF_4_, NaEuF_4_, LaF_3_, LiYF_4_, LiLuF_4_, BaYF_5_, BaGdF_5_) and oxides (e.g. Y_2_O_3_, ZrO_2_, GdVO_4_)^59,61^. We find that these other typical hosts also have fairly similar, low maximum phonon energies (≤600 cm^−1^) that favor radiative relaxation, for example ~550 cm^−1^ for Y_2_O_3_, ~500 cm^−1^ for ZrO_2_, and ~350 cm^−1^ for many of the commonly used fluorides^61,62^.

## Discussion

We investigated the effects of high excitation intensities on the two common luminescence thermometry signals for individual NaYF_4_:Yb^3+^,Er^3+^ nanoparticles. The widely used ratiometric method implies an apparent self-heating artifact of ~6 × 10^10^ K W^−1^, and two sets of luminescence lifetime thermometry measurements based on different excited states confirm the magnitude of this apparent thermal resistor. However, experiments that more directly manipulate the thermal environment conclusively demonstrate that the apparent temperature rise is not a result of poor heat dissipation from the nanoparticle to its surroundings. Instead, rate equation modeling shows that the increase in the luminescence intensity ratio with excitation intensity can largely be explained by a non-Boltzmann distortion of the population distribution of the green-emitting levels caused by population of the ^2^H_11/2_ state via multiphonon relaxation from higher-lying energy levels, which become heavily occupied at high-excitation intensities, as well as an increase in green photon-emitting transitions from highly excited Er^3+^ states. Although these modeling results cannot be directly applied to our excitation intensity-dependent lifetime measurements, the modeled intensity-dependent photophysics will almost certainly affect the lifetimes as well. We note that others have attributed related changes in the spectra^63^ or luminescence lifetimes^64^ of Yb^3+^/Er^3+^ co-doped materials to a broad class of effects involving impeded thermalization of a large non-equilibrium phonon distribution, frequently referred to as a “phonon bottleneck”. Yet the interpretation of such results remains far from fully settled, with recent studies^65^ disputing some of the earlier works and instead attributing the results to nanoparticle heating. Such effects are also expected to occur primarily at cryogenic temperatures and for nanoparticles dimensions of ~10 nm or smaller, and we thus believe that such effects are unlikely to play a large role in our measurements. We also explored the effects of modulated laser excitation on the measured temperature rise and reveal additional artifacts unique to this mode of operation that must also be considered in the context of ratiometric temperature measurements. All the effects we report have important practical implications for single-particle luminescence thermometry, but once understood can be calibrated or otherwise avoided, and thus we do not believe they limit the applicability of this technique. There exist many possible strategies for mitigating these artifacts, such as using relatively low-excitation intensities and performing both the initial calibration and all further measurements at a single intensity and frequency.

## Methods

### Nanoparticle sample preparation

NaYF_4_:Yb^3+^,Er^3+^ nanoparticles were dispersed in toluene and subsequently spin coated onto borosilicate glass, sapphire, or polyester substrates. Before spin coating, the borosilicate glass and sapphire substrates were sonicated for 10 min each in acetone, isopropyl alcohol, and deionized water, follow by 30 s of plasma cleaning. The polyester substrates were sonicated for 10 min each in acetone, isopropyl alcohol, ethanol, and deionized water before spin coating.

### Microscopy apparatus and single-particle identification

A custom scanning confocal microscopy apparatus was used to image the nanoparticles and obtain single-particle spectra. A 980 nm laser (Thorlabs TCLDM9 with a 300 mW diode) served as the excitation source and a 0.8 NA, ×100 air objective was used for all measurements. This objective also collected the emitted light and an 890 nm shortpass filter (Chroma) was used to remove residual laser light. A histogram of luminescence intensities from ~300 emission spots was used to determine the characteristic single particle intensity (Supplementary Fig. 1). TEM images were used to verify the morphology of the particles (Supplementary Fig. 2). Temperature calibration measurements were performed using a custom thermal stage able to control the sample temperature between ambient and 400 K (stability ± 1 K). For lifetime measurements, the output of the 980 nm laser was modulated using a function generator, and a TCSPC (PicoQuant PicoHarp 300) was used tag the arrival times of collected photons with respect to the modulated laser output. The time-resolved luminescence data was subsequently fit to an exponential decay. To isolate emission from only the green or red spectral bands, a 540 ± 20 or 650 ± 20 nm bandpass filter was used, respectively. To obtain the data shown in Fig. 6d, the output of the 980 nm laser was modulated using a function generator and the resulting emission was sent to a spectrometer.

### Polystyrene coatings

To obtain the polystyrene coated samples used for Fig. 4b, polystyrene pellets were dissolved in toluene to create a 15 mg/ml solution. This solution was subsequently drop cast or spin coated onto already prepared samples consisting of dispersed nanoparticles on borosilicate glass substrates.

## Electronic supplementary material

Supplementary Information

## Data Availability

The data that support the findings of this work are available from the authors upon reasonable request.
