# Supplementary material for: Apparent self-heating of individual upconverting nanoparticle thermometers
Source: Nat Commun. 2018 Nov 21;9:4907. doi: 10.1038/s41467-018-07361-0 (PMC6249317; doi:10.1038/s41467-018-07361-0)
Supplement: Supplementary file 1 — Supplementary Information [file 41467_2018_7361_MOESM1_ESM.pdf]

Supplementary Information: Apparent Self-Heating of Individual Upconverting Nanoparticle Thermometers

Andrea D. Pickel[1], Ayelet Teitelboim[2], Emory M. Chan[2], Nicholas J. Borys[2], P. James Schuck[2,3], and Chris Dames[1,2,*]

[1]Department of Mechanical Engineering, University of California, Berkeley, California 94720, United States

[2]The Molecular Foundry, Lawrence Berkeley National Laboratory, Berkeley, California 94720, United States

[3]Department of Mechanical Engineering, Columbia University, New York, New York 10027, United States

*cdames@berkeley.edu

Supplementary Note 1: Estimating R_{internal}, $R_{\text{substrate}}$, and R_{contact}

In our thermal model, we assume that the entire nanoparticle is at a uniform temperature T_{NP}. To validate this "lumped" assumption, we estimate an effective internal thermal resistance for the nanoparticle and compare this value to the largest external resistor, R_{air}. While the thermal resistance concept technically is not applicable in this case because the internal energy generation is volumetrically distributed throughout the particle, rather than occurring at a single point location, by approximating the nanoparticle as a sphere of diameter D with spatially uniform heat generation, we can define a suitable effective resistor with the correct K W^{-1} units – namely, the difference between the central and surface temperatures divided by the heat loading, which gives $R_{internal} = 1/(4\pi k_{particle}D)$. We take a conservatively low value of $k_{\text{particle}} \approx 1$ W/m-K for the nanoparticle thermal conductivity[1]. Thus, for a 50 x 50 x 50 nm^3 particle, $R_{\text{int}} \approx 2 \times 10^6$ K W^{-1}. Therefore, the Biot number $Bi = R_{\text{internal}}/R_{\text{air}}$ is ~$10^{-3} << 1$ and internal temperature gradients can be safely neglected to an excellent approximation.

$R_{\text{substrate}}$ is estimated as $1/(2k_{substrate}D)$ by approximating the particle as a heated disk on a semi-infinite medium, where $k_{\text{substrate}}$ is the substrate thermal conductivity. Because the majority of our experiments employ faceted 50 x 50 x 50 nm^3 nanorods with their hexagonal faces laying flat on a substrate, we take D to be 50 nm. Here, we use the bulk value of $k_{\text{glass}} \approx 1$ W m^{-1} K^{-1} and ignore possible phonon size effects, because we expect the average diameter of the contact area to be larger than the ~1 nm average phonon mean free path for glass at room temperature[2]. Under these approximations, we calculate $R_{\text{substrate}} \approx 1 \times 10^7$ K W^{-1} for a 50 x 50 x 50 nm^3 particle on a borosilicate glass substrate (the primary experimental configuration in this work). Thus although we neglect $R_{\text{substrate}}$ for the purpose of our conservative thermal estimate, in reality the substrate may act as a significant heat sink, which if accounted for in this analysis can only make the particle colder.

R_{contact} is the most challenging resistor to estimate due to the unknown contact area and limited theoretical understanding of thermal transport at nanoparticle-substrate interfaces. Calculated values for a 1 μm diameter Si nanowire on Si substrate surrounded by air[3]

range from ~10^4-10^8 K W^{-1}. Similarly, room temperature experimental values for carbon nanofibers and multiwalled carbon nanotubes on various substrates[4,5] are between ~10^4-10^7 K W^{-1}. If $R_{contact}$ in our experiments is on the high end of the range of reported values for these nanostructures with similar characteristic lengths, then $R_{contact}$ is comparable to R_{air} and a significant portion of the heat will be dissipated through the air. A very large $R_{contact}$ would not change the estimated thermal resistance or temperature rise, since in this case $R_{substrate} + R_{contact} >> R_{air}$ holds true. The magnitude of $R_{contact}$ does impact the anticipated effects of changing other components of the thermal circuit. For example, if $R_{contact}$ is the limiting resistance, then we expect the apparent temperature rise to be insensitive to changes in $R_{substrate}$, as discussed in the main text. If $R_{contact}$ is instead on the lower end of the range, a significant portion of the heat may flow through the contact. If $R_{substrate}$ is also small compared to R_{air} (i.e. now $R_{air} >> R_{substrate} + R_{contact}$), R_{model} and thus the estimated temperature rise can only decrease.

Supplementary Note 2: Single particle identification and characterization

To identify individual nanoparticles with characteristic dimensions far smaller than the diffraction-limited laser beam diameter, we followed a well-established statistical approach[6,7] in order to determine the characteristic brightness of a single particle. We obtained an APD scan that contains a total of approximately 300 luminescent emission spots. Supplementary Figure 1 shows a histogram of this data for the 50 x 50 x 50 nm^3 particles and the inset shows a representative portion of the APD scan. The characteristic brightness of a single particle can clearly be identified as ~6.5 kcnts per s. Given this information, when a new sample from the same batch of particles is imaged under the same conditions, we can rapidly identify single particles to use for subsequent thermal and intensity-dependent measurements. Supplementary Figure 1 also shows that the vast majority of the luminescent spots correspond to single nanoparticles, with a very small number of higher-intensity spots (> 10 kcnts/s) representing nanoparticle clusters, indicating that nanoparticle aggregation during the sample preparation process is minimal.

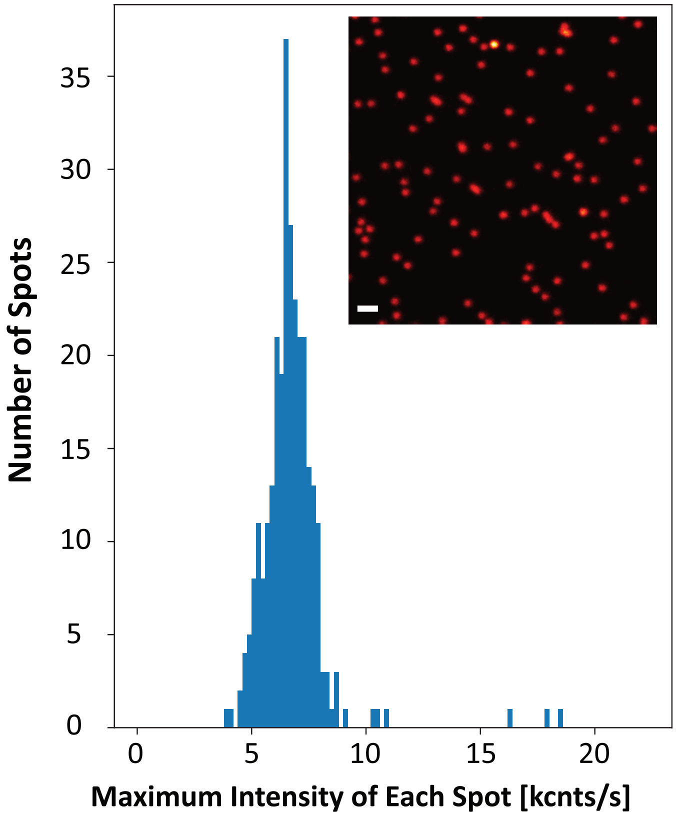

Supplementary Figure 1: Single-Particle Identification. Histogram of emission intensities for approximately 300 luminescent emission spots. The characteristic brightness of a single nanoparticles is clearly observed to be ~6.5 kcnts/s. The vast majority of the luminescent spots correspond to single particles. The inset shows a representative portion of the APD scan used to generate the histogram. Scale bar, 2 μm.

To assess the morphology and confirm the size of the 50 x 50 x 50 nm^3 particles, we obtained the transmission electron microscope (TEM) images of the nanoparticles shown in Supplementary Figure 2(a) and (b). Some of the particles are oriented such that their hexagonal faces can be observed, while particles that appear rounded or rectangular are lying on those sides. Supplementary Fig. 2(c) shows a TEM image of the 20 x 20 x 40 nm^3 particles[7].

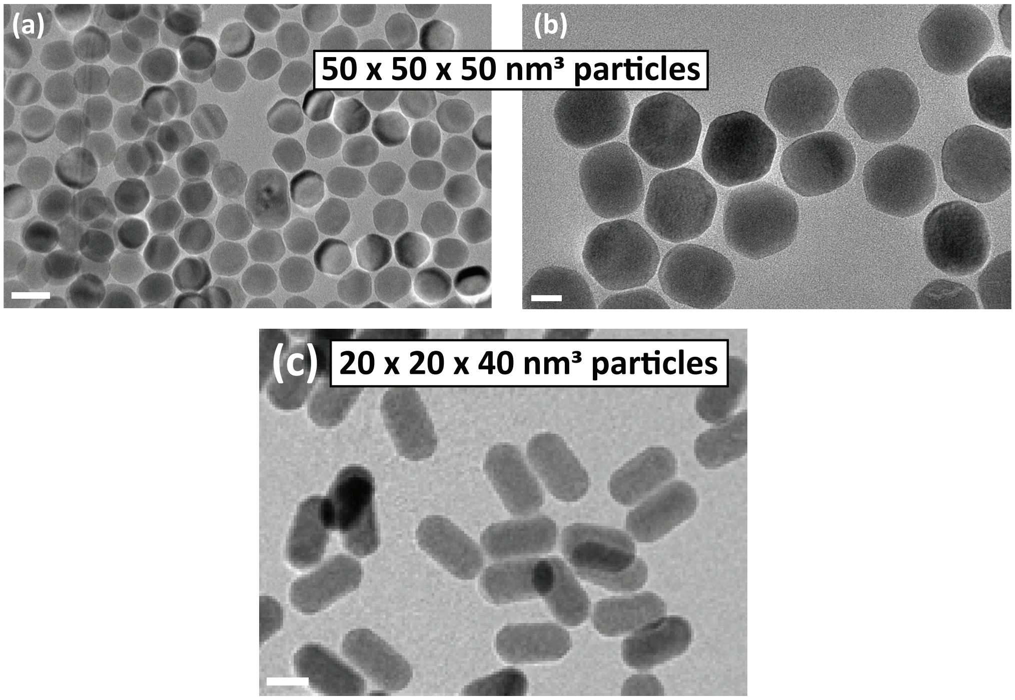

Supplementary Figure 2: TEM images of nanoparticles. (a) TEM image of hexagonally faceted 50 x 50 x 50 nm^3 particles. Scale bar, 50 nm. (b) Similar but at higher magnification. Scale bar, 20 nm. (c) TEM image of hexagonally faceted 20 x 20 x 40 nm^3 particles (reproduced from our previous work[7] with permission from the Royal Society of Chemistry). Scale bar, 20 nm.

Supplementary Note 3: Anomalous power dependence of *r* at low excitation intensities due to 556 nm peak

The green spectral emission of $NaYF_4:Yb^{3+},Er^{3+}$ UCNPs spans a wavelength range of approximately 515-565 nm. For the purpose of ratiometric thermometry, this emission band is typically separated into two sub-bands, a high-energy band of 515 nm < λ < 535 nm and a low energy band of 535 nm < λ < 560 nm. The high-energy band represents emission due to the $^2H_{11/2}$ to $^4I_{15/2}$ transition and the low-energy band represents emission due to the $^4F_{9/2}$ to $^4I_{15/2}$ transition. As noted in the main text, we exclude the peak observed at approximately 556 nm because of its known non-thermal origin. If instead that 556 peak is included in the second, low-energy band, the luminescence intensity ratio *r* displays a non-monotonic dependence on excitation intensity. As shown in

Supplementary Fig. 3(a), if the 556 nm peak (defined as the wavelength band between 548 and 560 nm) is included, r initially drops, and then begins increasing at an excitation intensity of roughly 5 x 10^4 W cm^{-2}. Supplementary Figure 3(b) shows an analogous plot obtained using the same experimental data if the ratio is instead defined to exclude the 556 nm peak (with a new wavelength cutoff of 548 nm), i.e. the same plot as Fig. 2(c) in the main text. Now r increases monotonically with excitation intensity, and the data is well described by a linear model. Using this approach, the mean and standard deviation for the fitted ΔE values (= 87.8 ± 0.8 meV) remain in good agreement with the theoretical range[8]. Supplementary Figure 3(c) shows the fraction of total counts that come from the wavelength band between 548 and 560 nm ($F_{(\lambda = 548 - 560\ \mathrm{nm})}$) as a function of excitation intensity. Below 5 x 10^4 W cm^{-2}, there is a sharp increase in $F_{(\lambda = 548 - 560\ \mathrm{nm})}$ with excitation intensity, followed by a plateau at higher excitation intensities. The initial jump in $F_{(\lambda = 548 - 560\ \mathrm{nm})}$ leads to the anomalous dependence of r on excitation intensity below 5 x 10^4 W cm^{-2}.

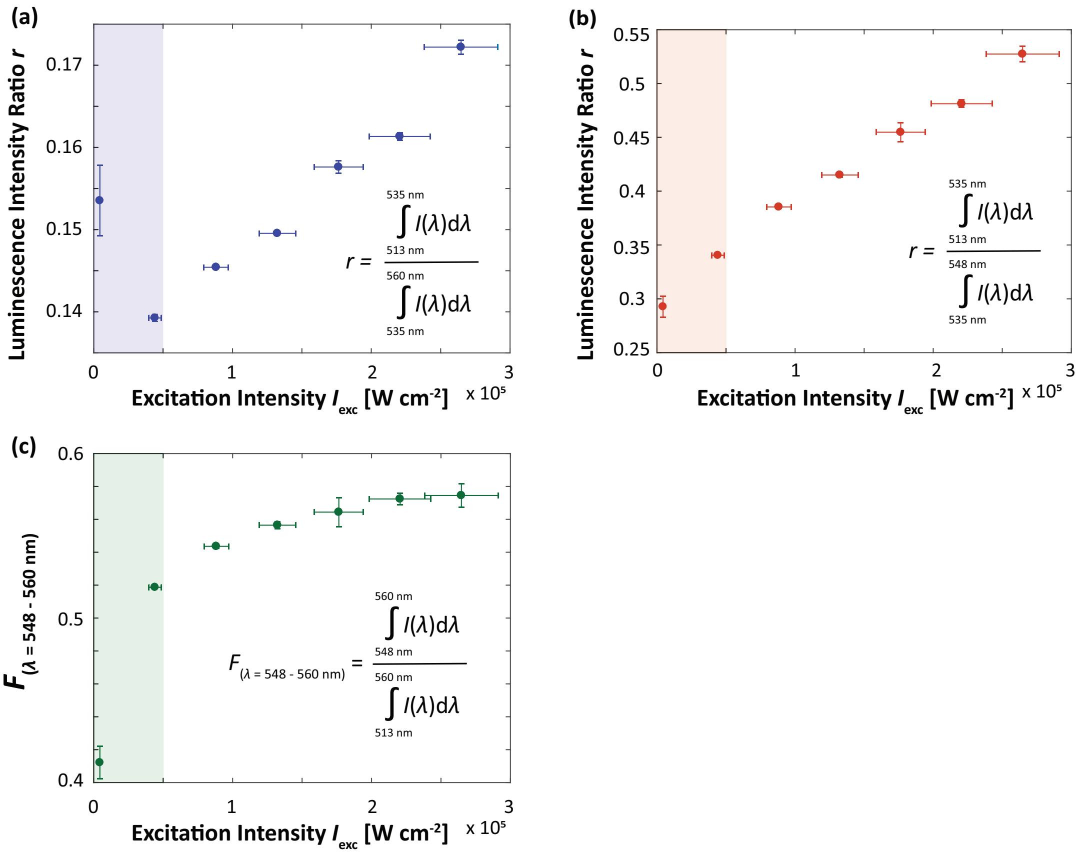

Supplementary Figure 3: Anomalous power dependence of *r*. (a) Luminescence intensity ratio as a function of excitation intensity if the 556 nm peak is included in the definition of the ratio. An anomalous power dependence is observed at excitation intensities below approximately 5 x 10^4 W cm^{-2} (shaded region). (b) When the 556 nm peak is excluded from the definition of *r*, we observe a linear increase in the ratio with excitation intensity across the entire excitation intensity range. This is the same data as Fig. 2(c) in the main text. (c) The anomalous low-excitation intensity behavior shown in part (a) corresponds to a sharp increase in the fraction of total counts coming from the wavelength band between 548 and 560 nm ($F_{(\lambda = 548 - 560\ \mathrm{nm})}$).

Supplementary Note 4: Error bars for ratiometric apparent temperature rise data

The error bars for all ratiometric apparent temperature rise data (i.e. Fig. 2(d) and Fig. 4(a), (b), and (c)) are calculated as follows. First, we account for the uncertainty introduced by using the calibrations of five different particles to estimate the apparent temperature rise measured by a sixth particle from the same batch. To do so, we consider

the five different slopes from fitting ln(r) vs. 1/T (a linear relationship obtained by taking natural log of Eq. (1) in the main text) for each particle shown in Fig. 2(a). Because we are interested in calculating apparent temperature rises (as opposed to absolute temperatures), it is the variation in these slopes that is important for our uncertainty analysis. Therefore, for every measured r value, the change in the ratio with respect to the zero-power value, i.e. $\Delta r = r - r_{\text{intrinsic}}(T = 296\text{ K})$, where $r_{\text{intrinsic}}(T = 296\text{ K}) \approx 0.289$ from Fig. 2(c), is calculated. Two consecutive spectra were taken at each I_{exc}, resulting in two Δr values for every I_{exc}. For each Δr, five different values of the apparent temperature rise are calculated using each of the five fitted slopes. This approach results in in ten apparent temperature rise values calculated at each I_{exc}. Finally, we plot the mean of these ten calculated values, and the error bars represent the standard deviation. Thus the error bars reflect both the variation between consecutive measurements and among particles in the same batch, which are the dominant sources of uncertainty in our measurements.

Supplementary Note 5: Error bars for luminescence lifetime apparent temperature rise data

The error bars for the luminescence lifetime apparent temperature rise data (Fig. 3(b) and (d)) are calculated in the same manner as the error bars for the ratiometric apparent temperature rise data, except that the underlying model is now a linear fit of τ_{lum} vs. T. For every measured τ_{lum} value, the change in the lifetime with respect to the zero-power value is calculated as $\Delta\tau_{\text{lum}} = \tau_{\text{lum}} - \tau_{\text{lum,intrinsic}}(T = 296\text{ K})$, where $\tau_{\text{lum,intrinsic}}(T = 296\text{ K})$ is obtained by extrapolating the $\tau_{\text{lum}}(I_{\text{exc}}, T)$ data at $T = 296$ K. We consider the variation in the slopes of the lines fitted to the lifetime vs. temperature data for the five particles shown in Fig. 3(a) and (c). Here, three consecutive measurements were performed at every I_{exc}, resulting in fifteen apparent temperature rise values for each I_{exc}. Again, the mean of the fifteen calculated values is plotted, and the error bars represent the standard deviation.

Supplementary Note 6: Rate Equation Modeling

Differential rate equations (DREs) were used to model steady-state changes in population n_i for each excited state i in the Yb^{3+} and Er^{3+} dopants. Our computational model[11,12]

solves systems of coupled DREs that describe the rate at which each lanthanide $4f^N$ manifold i is populated and depopulated by photon absorption, luminescence, energy transfer, and multiphonon relaxation. Supplementary Figure 4(a) displays the shift in population to higher-lying Er^{3+} energy levels as the excitation intensity is increased, and Supplementary Fig. 4(b) shows the resulting spectral emission changes with excitation intensity. As indicated by the magenta dashed lines in Supplementary Fig. 4(b), the wavelength bands used to calculate the modeled luminescence intensity ratio are 515-535 nm and 535-555 nm. The wavelength cutoff for the second band is slightly higher than the corresponding experimental value of 548 nm to account for the fact that the experimental spectral peaks have finite widths, in contrast with the simulations. Thus, although the experimental emission spectra are integrated only to 548 nm, some of this integrated emission intensity comes from transitions centered at slightly longer wavelengths. Supplementary Fig. 4(c) quantifies the increase in emission originating from the population change in the $^2H_{11/2}$ manifold relative to the $^4S_{3/2}$ manifold as I_{exc} is increased. Supplementary Fig. 4(d) shows how energy flow from higher-lying energy levels drives this increase and enumerates the energy level indices i used to identify the 36 DREs used in this work.

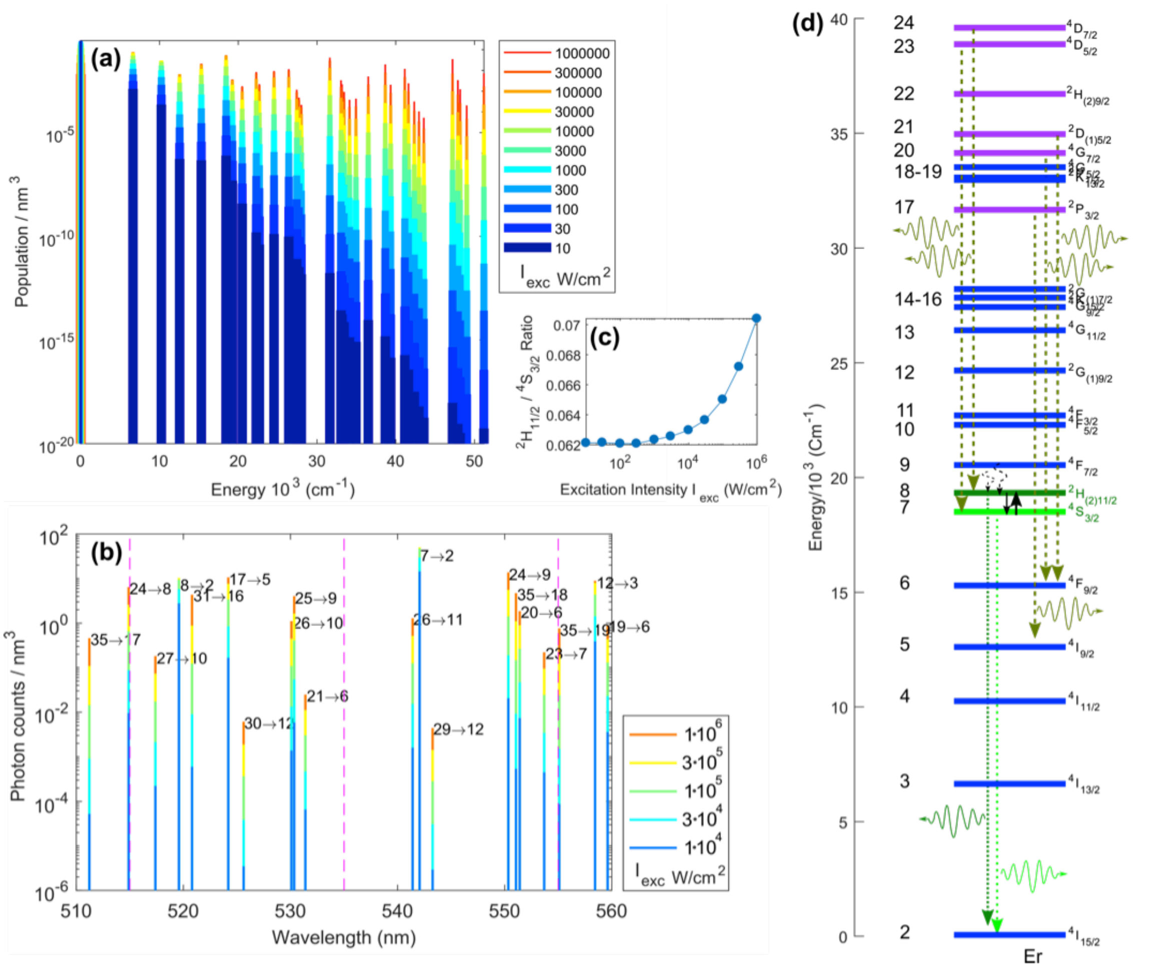

Supplementary Figure 4: Rate-equation modeling. (a) Spectral energy population of Er^{3+} levels for different excitation intensities of ~10^1-10^6 W cm $^{-2}$. Energy is presented in cm^{-1} relative to the ground state energy, which was fixed to zero. (b) Luminescence lines of Er^{3+} as a function of excitation intensity. Each emission line is labeled with its corresponding energy level transition (each *i* → *j* denotes initial to final). The magenta dashed lines depict the wavelength bands used to calculate the luminescence intensity ratio (515-535 nm and 535-555 nm). The transitions corresponding to relaxation from the $^2H_{11/2}$ and $^4S_{3/2}$ levels to the ground state are *8* → *2* and *7* → *2*, respectively. (c) Population ratio of levels $^2H_{11/2}$ / $^4S_{3/2}$ as a function of excitation intensity. (d) Energy level schematic of Er^{3+} with prominent luminescence radiative lines that contribute to emission in the wavelength bands shown in *(b)*. The transitions from the $^2H_{11/2}$ and $^4S_{3/2}$ levels to the ground state (*8* → *2* and *7* → *2)* are depicted in dark green and bright green,

respectively. Olive green transitions depict some contributions from higher-level excited states to the intensity ratio.

Supplementary Note 7: Solution of governing ODE for frequency-dependent temperature rise

Here we present the derivation of the modeled curves plotted in Fig. 6(c) of the main text The steady-periodic solution to the governing differential equation for the frequency-dependent temperature rise (main text Eq. (4)) can be obtained analytically using several different approaches. Here, we present a piecewise solution that takes advantage of the periodicity of Q(t). We start by defining the period of excitation, P, as $P = 1/f_{exc}$. For times t such that $NP < t < NP + P/2$, where $N = 0, 1, 2...$, $Q(t)$ has a constant value of Q_{max}. For times t such that $NP + P/2 < t < (N+1)P$, $Q(t)$ has a constant value of 0. Implicitly, this analysis assumes that the rise and fall time of $Q(t)$ is much faster than $\tau_{thermal}$ or τ_{lum}. The governing equation can therefore be written as:

$$\begin{aligned} Q_{max} - \frac{\theta(t)}{R_{thermal}} &= \rho c V \frac{d\theta(t)}{dt}, \qquad NP < t < NP + P/2 \\ -\frac{\theta(t)}{R_{thermal}} &= \rho c V \frac{d\theta(t)}{dt}, \qquad NP + P/2 < t < (N+1)P \end{aligned} \tag{1}$$

Both parts of Supplementary Equation (1) can be solved analytically using standard approaches. Since $\theta(t)$ must be continuous and has period P, the solutions to both parts of Supplementary Equation (1) must equal each other at $t = NP + P/2$, and also at $t = (N+1)P$. Because we are interested in the steady-periodic solution, N does not matter and is henceforth set to zero. The resulting piecewise solution to Supplementary Equation (1) can be expressed as follows:

$$\theta(t) = \begin{cases} Q_{max}R_{thermal}\left(1 + \dfrac{exp\left(-\dfrac{t}{\tau_{thermal}}\right)\left(exp\left(-\dfrac{t}{2\tau_{thermal}}\right) - 1\right)}{1 - exp\left(-\dfrac{t}{\tau_{thermal}}\right)}\right), & 0 < t < P/2 \\ Q_{max}R_{thermal}\dfrac{exp\left(-\dfrac{t}{\tau_{thermal}}\right)\left(exp\left(\dfrac{t}{2\tau_{thermal}}\right) - 1\right)}{1 - exp\left(-\dfrac{t}{\tau_{thermal}}\right)}, & P/2 < t < P \end{cases} \tag{2}$$

An analogous governing equation can be written for the luminescence response. I_{lum} increases with I_{exc}, which is here a square wave with period P. The response of I_{lum} to a step change in I_{exc} is taken to be a first-order exponential relaxation, with a time constant τ_{lum}. The governing equation for $I_{\text{lum}}(t)$ can thus be written as follows:

$$\begin{aligned} I_{lum,max} - I_{lum}(t) &= \tau_{lum}\frac{dI_{lum}(t)}{dt}, \qquad 0 < t < P/2 \\ -I_{lum}(t) &= \tau_{lum}\frac{dI_{lum}(t)}{dt}, \qquad P/2 < t < P \end{aligned} \tag{3}$$

By applying analogous stitching conditions every half period, the solution to Supplementary Equation (3) can be expressed as:

$$I_{lum}(t) = \begin{cases} I_{lum,max}\left(1 + \dfrac{exp\left(-\frac{t}{\tau_{lum}}\right)\left(exp\left(-\frac{t}{2\tau_{lum}}\right) - 1\right)}{1 - exp\left(-\frac{t}{\tau_{lum}}\right)}\right), & 0 < t < P/2 \\ I_{lum,max}\dfrac{exp\left(-\frac{t}{\tau_{lum}}\right)\left(exp\left(\frac{t}{2\tau_{lum}}\right) - 1\right)}{1 - exp\left(-\frac{t}{\tau_{lum}}\right)}, & P/2 < t < P \end{cases} \tag{4}$$

In this analytical framework, we assume that both τ_{lum} and $I_{\text{lum,max}}$ are constant, although in reality we observe that these quantities are modest functions of temperature. Figure 2(a) shows that the τ_{lum} decreases from approximately 260 μs to 180 μs between 296 K and 400 K, or roughly 3000 ppm K^{-1}. Accounting for this effect induces a negligible shift in the characteristic frequency at which the apparent temperature rise begins to drop off as compared to the curves shown in Fig. 6(c). We also observe from our experiments that $I_{\text{lum,max}}$ decreases as a function of temperature. At steady state, this is equivalent to approximating $I_{lum} = \gamma \cdot I_{exc}$, where we take γ to be independent of T. In reality, γ depends weakly on T, but this effect (also ~3000 ppm K^{-1}) is negligible compared to the much larger contrast between I_{exc} in the on and off states and thus has a similarly minimal impact on the modeled curves shown in Fig. 6(c). Consequently, we neglect these two second-order effects so that we can obtain an analytical solution for $\theta_{\text{measured}}(f_{\text{exc}})$.

In the main text, we qualitatively describe the physical phenomena that lead to a loss of sensitivity to R_{thermal} when $\tau_{\text{lum}} >> \tau_{\text{thermal}}$. Here, we provide a more detailed, quantitative explanation of their effect on θ_{measured}. The measurement is easiest to understand when $\tau_{\text{lum}} \rightarrow 0$, such that luminescence is only emitted during the half cycle when the excitation laser is on, at an approximately constant value $I_{\text{lum,on}}$. Again, $I_{\text{lum}}(t)$ is approximately a square wave between 0 and $I_{\text{lum,max}}$, synchronized with the $I_{\text{exc}}(t)$ square wave between 0 and $I_{\text{exc,on.}}$ Eq. (5) in the main text then simplifies to

$$\theta_{measured,\tau_{lum}\rightarrow 0} = 2f_{exc}\int_0^{\frac{1}{2f_{exc}}} \theta(t)dt \tag{5}$$

As discussed in the main text, here $\theta_{measured,\tau_{lum}\rightarrow 0}$ is an average of the true $\theta(t)$ but averaged only over the half cycle when I_{exc} is on. If $\tau_{\text{thermal}} << 1/f_{\text{exc}}$, this will simply yield the DC value, θ_{DC}. This θ_{DC} is an important reference value for other regimes. For example, if $\tau_{\text{thermal}} >> 1/f_{\text{exc}} >> \tau_{\text{lum}}$, the temperature cannot keep up with the laser heating oscillations and instead $\theta(\text{t}) \rightarrow 0.5\theta_{\text{DC}}$, so $\theta_{\text{measured}} = 0.5\theta_{\text{DC}}$ (Fig. 6(b)). Thus, for $\tau_{\text{lum}} \rightarrow 0$, the frequency at which θ_{measured} transitions from θ_{DC} to $0.5\theta_{\text{DC}}$ corresponds to $f_{\text{exc}}\tau_{\text{thermal}} \sim 1$.

However, in the actual experimental regimes of greatest interest, we find that τ_{lum} cannot simply be set to 0. Indeed, the most significant consequence of Eq. (5) occurs when $\tau_{\text{lum}} >> 1/f_{\text{exc}} >> \tau_{\text{thermal}}$. In this regime, the spectrometer spectral ratio will also correspond to $\theta_{\text{measured}} \approx 0.5\theta_{\text{DC}}$, even though in reality $\theta(t)$ remains a square wave between 0 and θ_{DC}. Due to the large τ_{lum}, $I_{\text{lum}}(t)$ now remains essentially constant throughout the cycle, and Eq. (5) simplifies to

$$\theta_{measured,\tau_{lum}\rightarrow \infty} = f_{exc}\int_0^{\frac{1}{f_{exc}}} \theta(t)dt \tag{6}$$

In contrast to Supplementary Equation (5), now $\theta_{measured,\tau_{lum}\to\infty}$ represents an average of the true $\theta(t)$ over the full cycle. Thus, even though the true θ in Fig. 6(a) still reaches θ_{DC}, the spectrometer ratio will correspond to $0.5\theta_{DC}$, but for a reason that has nothing to do with $f_{exc}\tau_{thermal}$ ~1. Here, this transition instead occurs when $f_{exc}\tau_{lum}$ ~1. As noted in the main text, the key takeaway is that there are two very different mechanisms that can cause a transition from $\theta_{measured} = \theta_{DC} \rightarrow 0.5\theta_{DC}$ with increasing f_{exc}, resulting in a loss of sensitivity to $R_{thermal}$ when $\tau_{lum} >> \tau_{thermal}$.

By substituting the appropriate expressions for $I_{lum}(t)$ and $\theta(t)$ (i.e. Supplementary Equation (2) and Supplementary Equation (4), respectively) into Eq. (5) and carrying out the integration, the analytical solution for $\theta_{measured}$ at any value of τ_{lum} can be expressed as

$$\theta_{measured}(f_{exc}) = Q_{max}R_{thermal}\Bigg[1 - 2f_{exc}\tau_{thermal}\cdot tanh\left(\frac{1}{4f_{exc}\tau_{thermal}}\right) - 2f_{exc}\tau_{lum}\cdot tanh\left(\frac{1}{4f_{exc}\tau_{lum}}\right) + \frac{f_{exc}\tau_{thermal}\,\tau_{lum}}{\tau_{thermal}+\tau_{lum}}\cdot \left(tanh\left(\frac{1}{4f\tau_{thermal}}\right) + tanh\left(\frac{1}{4f\tau_{lum}}\right)\right)\Bigg]. \quad (7)$$

This analytical solution is plotted for different values of $R_{thermal}$ in Fig. 6(c) in the main text.

Supplementary Note 8: Confirmation of successful laser modulation up to 10 kHz

For the modulated excitation ratiometric measurements described in the main text, the output of the 980 nm laser diode was modulated using a function generator. One potential concern is that the laser output may not be successfully modulated at high frequencies. To ensure that the modulated laser output still resembled a square wave at the highest frequencies used in our experiments, we used an avalanche photodiode (APD) to measure the 980 nm signal reflected from a silicon wafer over one excitation period for frequencies between 10 Hz and 100 kHz, as shown in Supplementary Figure 5. Above 10 kHz, the laser output begins to deviate significantly from the expected square wave shape, and we thus exclude data taken at frequencies above 10 kHz.

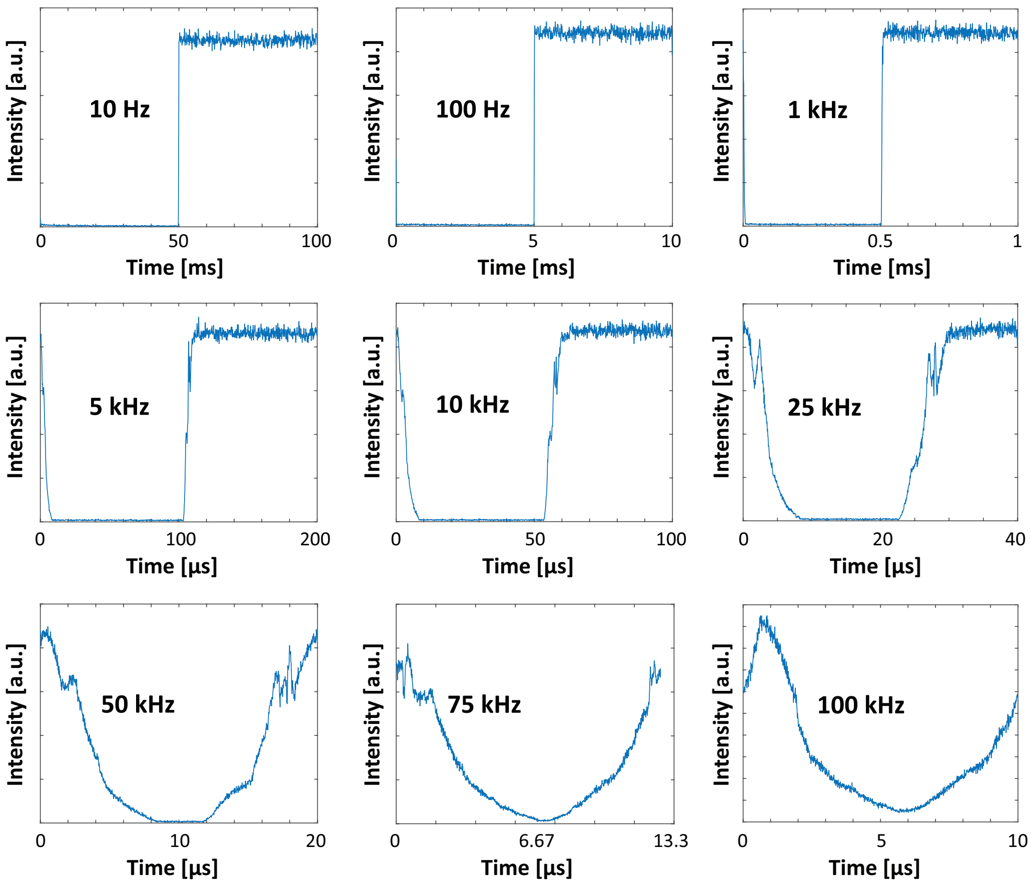

Supplementary Figure 5: Confirmation of laser modulation. All plots show the laser intensity as a function of time over a single excitation period, at the frequencies given on each plot. These results demonstrate that the output of the 980 nm laser diode can be successfully modulated using a function generator up to frequencies of approximately 10 kHz. We exclude data taken at frequencies above 10 kHz since the laser output deviates significantly from a square wave.

Supplementary Note 9: Error bars for modulated excitation ratiometric data

The error bars for the experimental data shown in Fig. 6(c) differ from the error bars for all other ratiometric data because, in this case, the ratio values are not converted to an apparent temperature rise. The final quantity that is plotted is $\theta_{\text{measured}}(f_{\text{exc}})/\theta_{\text{measured}}(f_{\text{exc}} = 0)$. Because the change in the ratio with respect to the zero-power value, i.e. $\Delta r = r - r_{\text{intrinsic}}(T = 296\text{ K})$, varies nearly linearly with θ over the temperature range of interest, $\theta_{\text{measured}}(f_{\text{exc}})/\theta_{\text{measured}}(f_{\text{exc}} = 0)$ can be well approximated as $\Delta r(f_{\text{exc}})/\Delta r(f_{\text{exc}} = 0)$. This

normalization requires choosing a value for $r(f_{exc} = 0)$. Because the low-frequency data points in Fig. 6(c) are somewhat noisy, $r(f_{exc} = 0)$ is estimated by taking the mean of the 11 lowest-frequency values. To account for the fact that changing this $r(f_{exc} = 0)$ normalization factor shifts the experimental data in Fig. 6(c), values of $r(f_{exc} = 0)$ one standard deviation above and below the mean were also used. Thus the error bars at each frequency represent the mean and standard deviation of the $r(f_{exc})/r(f_{exc} = 0)$ values calculated for each of three consecutive trials using three different values of $r(f_{exc} = 0)$ for each trial.

Supplementary Note 10: Apparent temperature rise of nanoparticle surrounded by water drop

In order to more directly assess the effects of manipulating R_{air}, we measured $r(I_{exc})$ for a nanoparticle surrounded by a drop of deionized water. To facilitate these measurements, we reversed the orientation of our nanoparticle sample such that the nanoparticles were on the top side of the coverslip and imaging was performed through the coverslip (left inset of Supplementary Figure 6). In this configuration, a scan with no water added revealed that there is some loss in emission intensity when imaging through the coverslip. This result is not unexpected, because our objective has no built-in coverslip correction and thus cannot compensate for the refractive index contrast between glass and air[9] (a coverslip-corrected objective was not available). Upon adding a drop of water to cover the same nanoparticle that was previously imaged through the coverslip, no further loss in emission intensity was observed. We were thus able to obtain auxiliary $r(I_{exc})$ data for a particle surrounded by water. The water drop was monitored throughout the experiment to ensure that it did not evaporate. A challenge, however, is that it is difficult to determine the true excitation intensity seen by the nanoparticle. While refraction aberrations will broaden the focal spot and reduce the local excitation intensity, we measure no significant difference in the total laser power when the laser beam passes through a coverslip due the large area of our optical power meter (incidentally confirming that absorption and reflection by the coverslip are negligible). Thus, we plot the r values as a function of the power measured at the entrance to the microscope. Supplementary Fig. 6 shows that the results for a nanoparticle in this configuration are similar regardless

of whether it is surrounded by air or water, despite the fact that water has a thermal conductivity more than an order of magnitude higher than that of air. Consequently, this measurement further strengthens the conclusion associated with Fig. 4(a) and (b), which is that none of the external thermal resistors controls the apparent temperature rise.

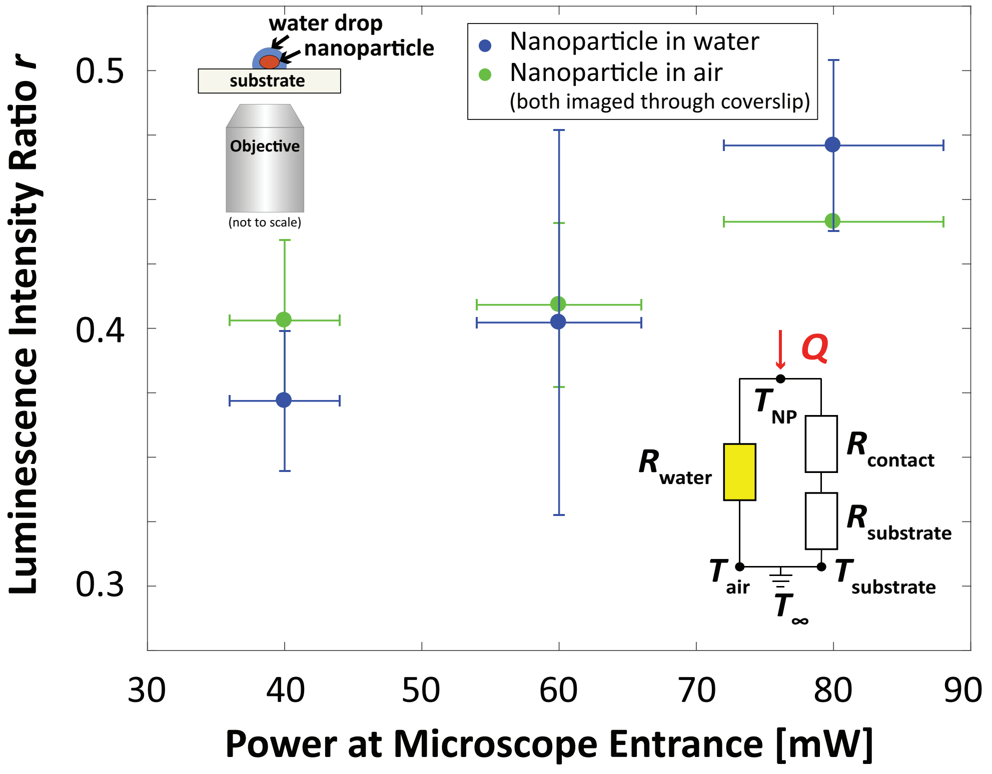

Supplementary Figure 6: Nanoparticle surrounded by water drop. Measured $r(I_{exc})$ for a nanoparticle surrounded by air and a nanoparticle surround by a drop of water, both imaged through a glass coverslip. The r values are similar regardless of whether the nanoparticle is surrounded by air or water, further confirming that none of the external thermal resistors controls the apparent temperature rise.

Supplementary Note 11: Temperature calibration and apparent temperature rise of nanoparticle ensembles

Here, we extend the results of Fig. 2(a) and (d) to small ensembles of nanoparticles. Although our nanoparticle samples have excellent colloidal stability and nanoparticle aggregates are thus rare, we located several cases in which two particles clustered

together. We measured the luminescence intensity ratio of two such double-particle clusters as a function of temperature and excitation intensity. For each double particle cluster, we also performed the same measurements on a nearby single particle. Supplementary Figure 7 shows that the apparent temperature rise of the double-particle clusters is essentially indistinguishable from that of the single particles within experimental noise. From a heat transfer perspective, the fact that the apparent temperature rise of the double-particle clusters and the single particles is the same further confirms that this effect is non-thermal. The emission intensity of the double-particle clusters is exactly twice that of the single particles (see insets of Supplementary Fig. 7(a)), suggesting that two particles are clustered together in the center of the laser spot. If the two particles were further apart, yet still both within the laser spot, we would expect the emission intensity to be notably less than twice that of a single particle due to the effective decrease in excitation intensity. The self-heating estimate for a double particle cluster is thus similar to that for a single particle, with the addition of a symmetry plane at the junction of the two particles, which can be treated as an adiabatic surface[10]. Consequently, we expect the temperature rise of a double particle cluster to be up to twice that of a single particle, yet Supplementary Fig. 7(b) shows an essentially identical apparent temperature rise in both cases. More broadly, this result suggests that the intensity-dependent photophysics we observe in this work for single particles should also be considered for ensembles.

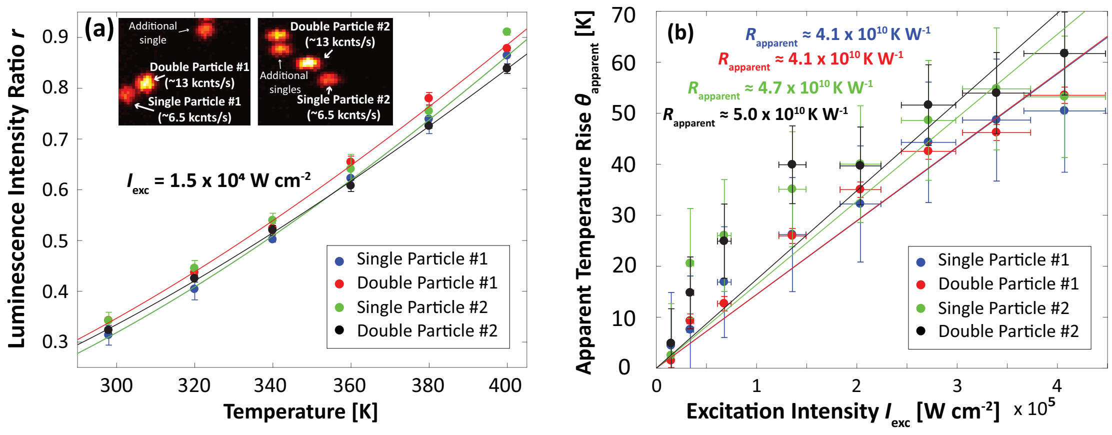

Supplementary Figure 7: Nanoparticle ensembles. (a) Ratio versus temperature calibration (I_{exc} = 1.5 x 10^4 W cm^{-2}) for two different double particle clusters and a nearby single particle in each case. The calibrations for the single particles and the double-particle clusters are nearly identical. The insets show avalanche photodiode (APD) scans of the double-particle clusters and nearby single particles. (b) At higher I_{exc} the apparent temperature rises of the same single particles and double-particle clusters are all quite similar, further indicating that this effect is not thermal in nature.

Supplementary References

1. Wang, L. *et al.* A New Cubic Phase for a $NaYF_4$ Host Matrix Offering High Upconversion Luminescence Efficiency. *Adv. Mater.* **27,** 5528–5533 (2015).
2. Chen, G. Nonlocal and Nonequilibrium Heat Conduction in the Vicinity of Nanoparticles. *J. Heat Transfer* **118,** 539–545 (1996).
3. Prasher, R. Predicting the Thermal Resistance of Nanosized Constrictions. *Nano Lett.* **5,** 2155–2159 (2005).
4. Warzoha, R. J. & Fleischer, A. S. Heat flow at nanoparticle interfaces. *Nano Energy* **6,** 137–158 (2014).
5. Yang, J. *et al.* Measurement of the intrinsic thermal conductivity of a multiwalled carbon nanotube and its contact thermal resistance with the substrate. *Small* **7,** 2334–2340 (2011).
6. Gargas, D. J. *et al.* Engineering bright sub-10-nm upconverting nanocrystals for single-molecule imaging. *Nat. Nanotechnol.* **9,** 300–305 (2014).
7. Kilbane, J. D. *et al.* Far-field optical nanothermometry using individual sub-50 nm upconverting nanoparticles. *Nanoscale* **8,** 11611–11616 (2016).
8. Zhou, S. *et al.* Upconversion luminescence of $NaYF_4$: Yb^{3+}, Er^{3+} for temperature sensing. *Opt. Commun.* **291,** 138–142 (2013).
9. Fellers, T. J. & Davidson, M. W. Coverslip Correction. *Nikon MicroscopyU*
10. Bergman, T. L., Lavine, A. S., Incropera, F. P. & DeWitt, D. P. *Fundamentals of Heat and Mass Transfer*. (2011).
11. Chan, E. M., Gargas, D. J., Schuck, P. J. & Milliron, D. J. Concentrating and recycling energy in lanthanide codopants for efficient and spectrally pure emission: The case of $NaYF_4$:Er^{3+}/Tm^{3+} upconverting nanocrystals. *J. Phys. Chem. B* **116,** 10561–10570 (2012).
12. Chan, E. M. Combinatorial approaches for developing upconverting nanomaterials: high-throughput screening, modeling, and applications. *Chem. Soc. Rev.* **44,** 1653–1679 (2015).
